# A newly isolated giant virus, ushikuvirus, is closely related to clandestinovirus and shows a unique capsid surface structure and host cell interactions

**DOI:** 10.1128/jvi.01206-25

**Published:** 2025-11-24

**Authors:** Jiwan Bae, Narumi Hatori, Raymond N. Burton-Smith, Kazuyoshi Murata, Masaharu Takemura

**Affiliations:** 1 Department of Mathematics and Science Education, Graduate School of Science, Tokyo University of Science, Tokyo, Japan; 2 Exploratory Research Center on Life and Living Systems, National Institute of Natural Sciences, Okazaki, Japan; 3 National Institute for Physiological Sciences, National Institutes of Natural Sciences, Okazaki, Japan; 4 Department of Physiological Sciences, The Graduate University for Advanced Studies (SOKENDAI), Okazaki, Japan; Michigan State University, East Lansing, Michigan, USA

**Keywords:** ushikuvirus, giant virus, virus isolation, vermamoeba, family *Mamonoviridae*, clandestinovirus, cytopathic effect, spike proteins, virus infection and proliferation, host-virus interaction

## Abstract

The family *Mamonoviridae*, assigned in 2023, consists of three strains of medusavirus that infect acanthamoeba. A closely related species, clandestinovirus, which infects vermamoeba, was reported in 2021. Here, we report a newly identified clandestinovirus-like virus, named ushikuvirus, isolated from a freshwater pond in Japan. The ushikuvirus genome was at least 666,605 bp and contained 784 genes. Annotation revealed that a substantial proportion (58%) of open reading frames (ORFs) are ORFans, and 25% of ORFs are similar to those of other viruses in the phylum *Nucleocytoviricota*. Among ORFs sharing sequence similarity with other viruses, a large proportion (80%) were similar to the clandestinovirus sequences. However, ushikuvirus shows several remarkable features. (i) The capsid surface has multiple spike-like “cap” structures, some of which exhibit a fibrous structure. (ii) The infection cycle is longer than that of medusavirus and clandestinovirus, and the virus exhibits a unique cytopathic effect (CPE) that causes enlargement of host vermamoeba cells. (iii) The virus forms a viral factory for duplication and destroys the nuclear membrane of vermamoeba cells, a phenomenon not observed with medusavirus and clandestinovirus. These characteristics indicate that this newly isolated giant virus related to the family *Mamonoviridae* and Clandestinovirus may represent a key taxon for elucidating virus-host interactions and the evolution of this virus group.

**IMPORTANCE:**

The family *Mamonoviridae* consists of only one genus, including three species: *Medusavirus medusae*, *Medusavirus sthenus*, and the recently described medusavirus euryale. These three medusaviruses have been reported to infect *Acanthamoeba castellanii*. Meanwhile, clandestinovirus, a closely related species in the family *Mamonoviridae*, infects vermamoeba. In these viruses, genome replication takes place in the nucleus of the host cell, and like eukaryotes, the genome encodes a full set of histones and has numerous spikes on the capsid surface. Here, we report a new member of this unique virus group, ushikuvirus, which displays distinct features including cytopathic effects in vermamoeba cells. These findings improve our understanding of the biological significance of the family *Mamonoviridae* and closely related taxa and provide a basis for elucidating the evolutionary relationships of giant viruses with their eukaryotic hosts.

## INTRODUCTION

Since the discovery of Acanthamoeba polyphaga mimivirus in 2003 (1), a wide variety of complex dsDNA viruses in the phylum *Nucleocytoviricota* have been isolated from water and soil environments worldwide (2–4). Several families whose hosts are mainly eukaryotic unicellular organisms (e.g., *Acanthamoeba* spp.) have been assigned to this large virus group, including the families *Mimiviridae*, *Allomimiviridae*, *Shizomimiviridae*, *Mesomimiviridae*, *Marseilleviridae*, *Pithoviridae*, and *Orpheoviridae*, in addition to the well-known virus families (documented since the 20th century) *Poxviridae*, *Ascoviridae*, *Asfarviridae*, *Phycodnaviridae*, and *Iridoviridae*, which have a wide range of hosts, from unicellular to multicellular eukaryotes (3).

In the virus taxonomy released in 2023 by the International Committee on Taxonomy of Viruses (ICTV), the family *Mamonoviridae* has been newly added to this large virus group (5). This family consists of a single genus *Medusavirus*, including *Medusavirus medusae*, *Medusavirus sthenus*, and a newly isolated putative third species, medusavirus euryale (6–8). The first strain in the genus *Medusavirus* was originally isolated from hot spring water in Hokkaido, Japan, in 2019 (*Acanthamoeba castellanii* medusavirus, species *Medusavirus medusae*), followed by the discovery of a strain isolated from a freshwater river in Kyoto, Japan, in 2021 (medusavirus stheno, species *Medusavirus sthenus*), respectively (6, 7). The genus *Medusavirus* has several characteristic features (6–8). The genomes encode a full set of histones (H2A, H2B, H3, H4, and linker histone H1), except for medusavirus euryale. Genomic DNA is replicated in the host cell nucleus, without constructing a virion factory in the cytoplasm, as observed in mimivirus and marseillevirus. The family B DNA polymerase gene is more similar to eukaryotic DNA polymerase δ than to B DNA polymerases of other viral families. Based on these properties and the ancestral features of the medusavirus genome, it has been proposed that the ancestor of medusavirus may have contributed to the emergence of eukaryotes (9). However, the genome of the recently isolated medusavirus euryale does not encode linker histone H1 (8). This report and a recent cryo-electron microscopy (cryo-EM)-based nucleosome reconstruction have revealed that the linker histone H1 of medusavirus does not function as a linker as in eukaryotes, indicating that the linker histone is not essential to the infection cycle of medusavirus (10).

In 2021, a *Mamonoviridae*-related giant virus named clandestinovirus was discovered from a wastewater sample in Saint-Pierre-de-Mézoargues, France (11). Clandestinovirus infects the unicellular eukaryote *Vermamoeba vermiformis*, not *Acanthamoeba* spp. The linear dsDNA genome of 581,987 bp contains 617 genes, exceeding the estimated number of genes for other members in the family *Mamonoviridae*. Although it is very closely related to the family *Mamonoviridae*, phylogenetically and its genomic DNA replicates in the host nucleus, similar to medusaviruses (6, 9), clandestinovirus is not included in the family *Mamonoviridae* because it shows substantial divergence at the intra- and inter-family levels from the other two *Mamonoviridae* species, *M. medusae* and *M. sthenus* (5). As mentioned previously, the infectivity of clandestinovirus to vermamoeba clearly distinguishes this taxon from the family *Mamonoviridae*, which infects acanthamoeba. To date, several giant viruses have been isolated by co-culture with vermamoeba, including faustovirus, kaumoebavirus, yasminevirus, orpheovirus, tupanvirus, and fadolivirus (11–18). It is possible that the attachment and invasion mechanisms of these giant viruses into vermamoeba differ from those of giant viruses infecting acanthamoeba, despite the fact that tupanvirus can infect both amoebae (16).

Here, we present a giant virus newly isolated from a freshwater pond in Ibaraki Prefecture near Tokyo, Japan, named ushikuvirus. The genomic, phylogenetic, and structural features of the virus are characterized as well as its cytopathic effects in vermamoeba cells. The newly identified features of ushikuvirus provide insights into the evolution of vermamoeba-infecting giant viruses and the mechanism underlying host-virus interactions.

## RESULTS

### Isolation of new giant virus

**Fig 1:**
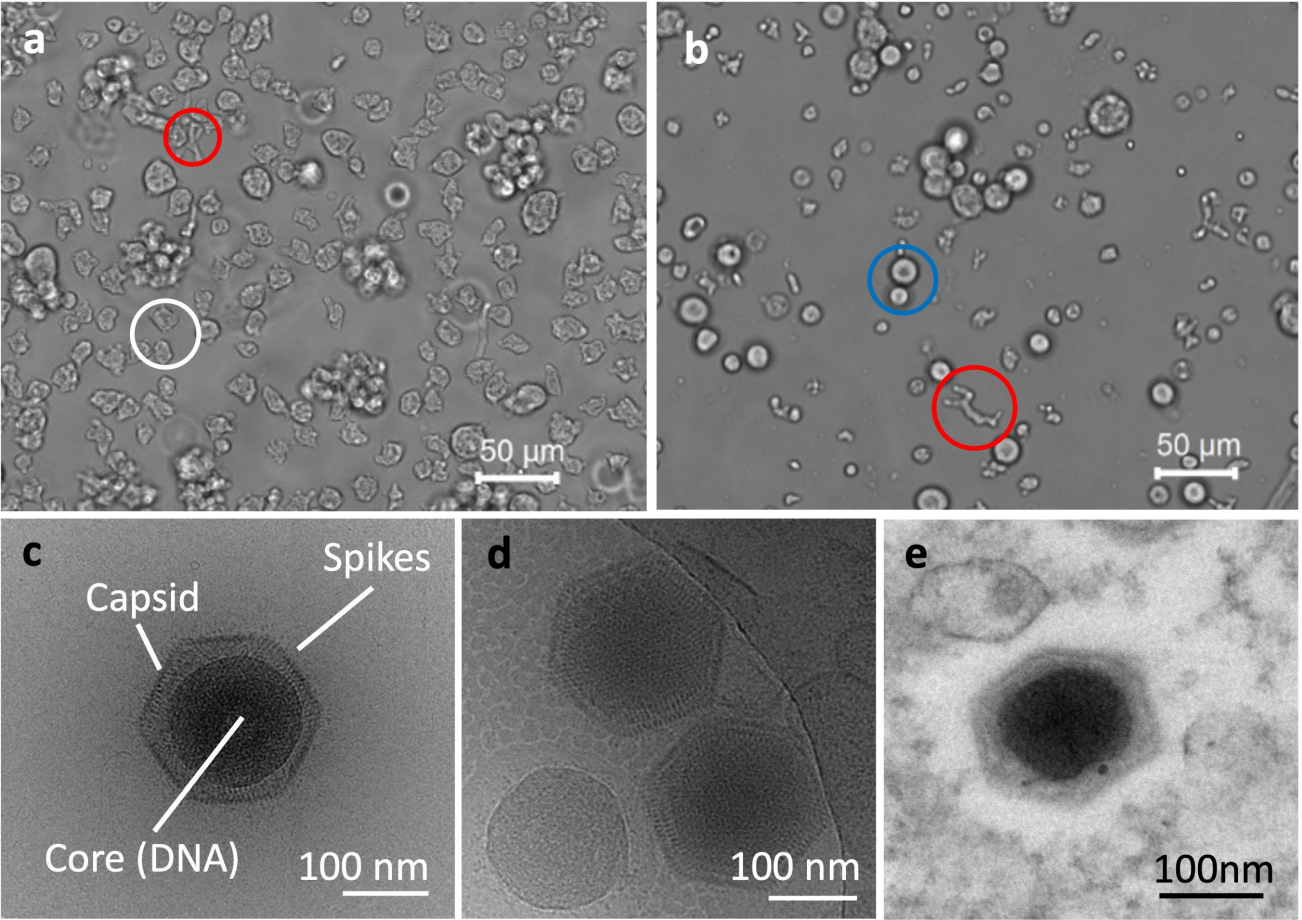
Morphological features of ushikuvirus particles and CPE of infected cells. (**a**) A phase-contrast microscope image of the non-infected vermamoeba cells. (**b**) A phase-contrast microscope image of the ushikuvirus-infected vermamoeba cells showing CPE at 5 dpi. Each circle represents a different phase of the cell: globular (white), fusiform (red), and rounded (blue) shapes. (**c, d**) Cryo-EM images of ushikuvirus particles. (**e**) A c-TEM image of the ushikuvirus particle in infected vermamoeba cells at 5 dpi. Scale bars: a and b, 50 nm; c, d, and e, 100 µm.

We isolated a new giant virus infecting *Vermamoeba vermiformis* from the freshwater pond "Ushiku-numa" in Ibaraki Prefecture near the Tokyo metropolitan area of Japan. This study reports, for the first time, a giant virus isolated from aquatic environments in Japan that infects Vermamoeba. *Vermamoeba vermiformis*, formerly known as *Hartmannella vermiformis*, belongs to the class *Tubulinea* (whereas *Acanthamoeba* spp. belongs to the class *Discosea*) and forms two shapes in the healthy state: globular and fusiform (Fig. 1a and b) (19). Under microbial infection, vermamoebae often change from globular to fusiform and finally form a rounded shape, as observed in the endosymbiotic bacterial *Candidatus* phylum *Dependentiae* (20). Similarly, after infection with ushikuvirus, vermamoebae showed a shape change from globular to partly fusiform and finally rounded (Fig. 1a and b).

### Morphological features of ushikuvirus

**Fig 2:**
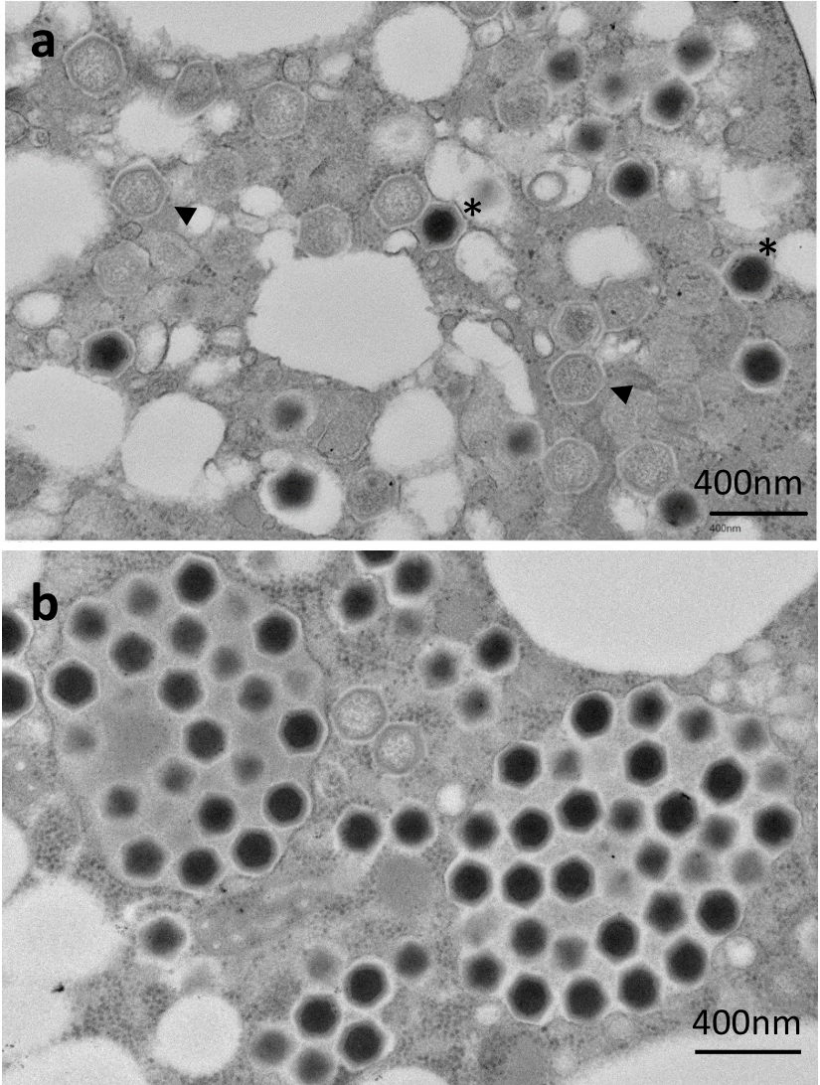
Morphological features of ushikuvirus particles in infected cell cytoplasm at 5 hpi revealed by c-TEM analysis. (**a**) Genome-containing (asterisks) and empty particles (arrowheads) were detected in the cytoplasm. (**b**) Numerous full particles were observed in the cytoplasm with or without surrounding membranes.

Cryo-transmission electron microscopy (cryo-TEM) and conventional transmission electron microscopy (c-TEM) revealed that ushikuvirus particles are morphologically very similar to medusavirus infecting acanthamoeba (6), with an icosahedral shape and numerous short spikes on the capsid surface. Excluding the surface spikes, the particle diameter was approximately 250 nm (Fig. 1c and d). After ushikuvirus infection, the vermamoebae exhibited a CPE, with cell rounding (Fig. 1b). Icosahedral-shaped virus particles were observed in the cytoplasm of the virus-infected round cells (Fig. 1e). In the cytoplasm of some vermamoeba infected with ushikuvirus at 5 hpi, virus particles with a core containing genomic DNA were observed in smaller numbers than those without a core (Fig. 2a), suggesting the early-stage cells of virus infection. In contrast, in the cytoplasm of some vermamoeba-infected ushikuvirus, virus particles with a core were observed in larger numbers than those without a core. Cryo-EM observations of released ushikuvirus particles revealed no empty particles outside the cells (Fig. 1c and d), suggesting that these clustered-filled viral particles represent the late stage of viral infection (Fig. 2b). In addition, at these late-stage cells, newly assembled viruses were accumulated and surrounded with a membrane (Fig. 2b), which have been observed in clandestinovirus and marseilleviruses (11, 21–23).

### Morphological change of vermamoeba under ushikuvirus infection

**Fig 3:**
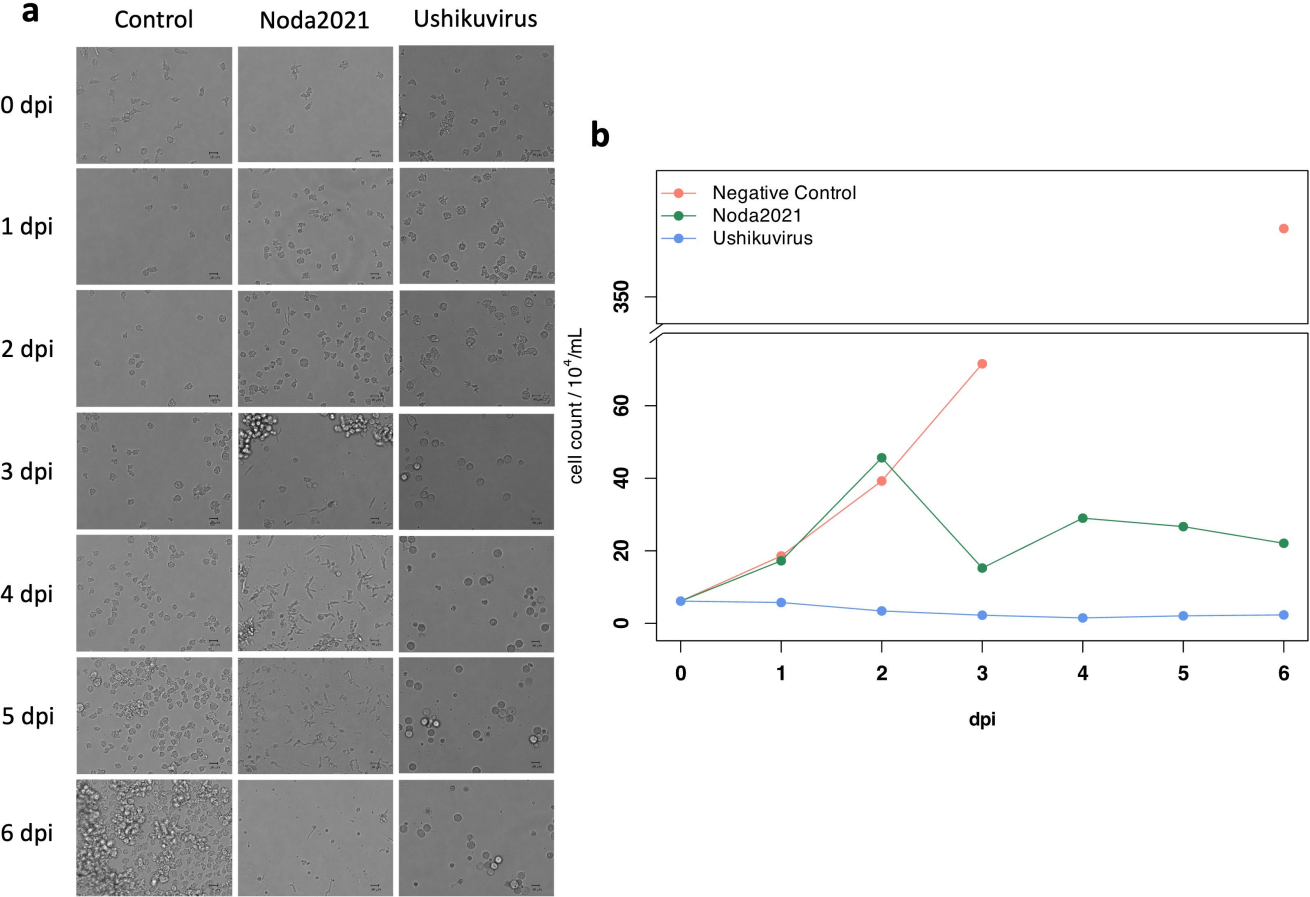
CPE of the host cell morphology by ushikuvirus infection. (**a**) Morphological changes of Noda2021-infected and ushikuvius-infected vermamoeba cells revealed by phase-contrast microscopy, from 0 to 6 dpi (days post-infection). (**b**) Cell counts and time-dependent changes in Noda2021-infected and ushikuvirus-infected vermamoeba cells.

**Fig 4:**
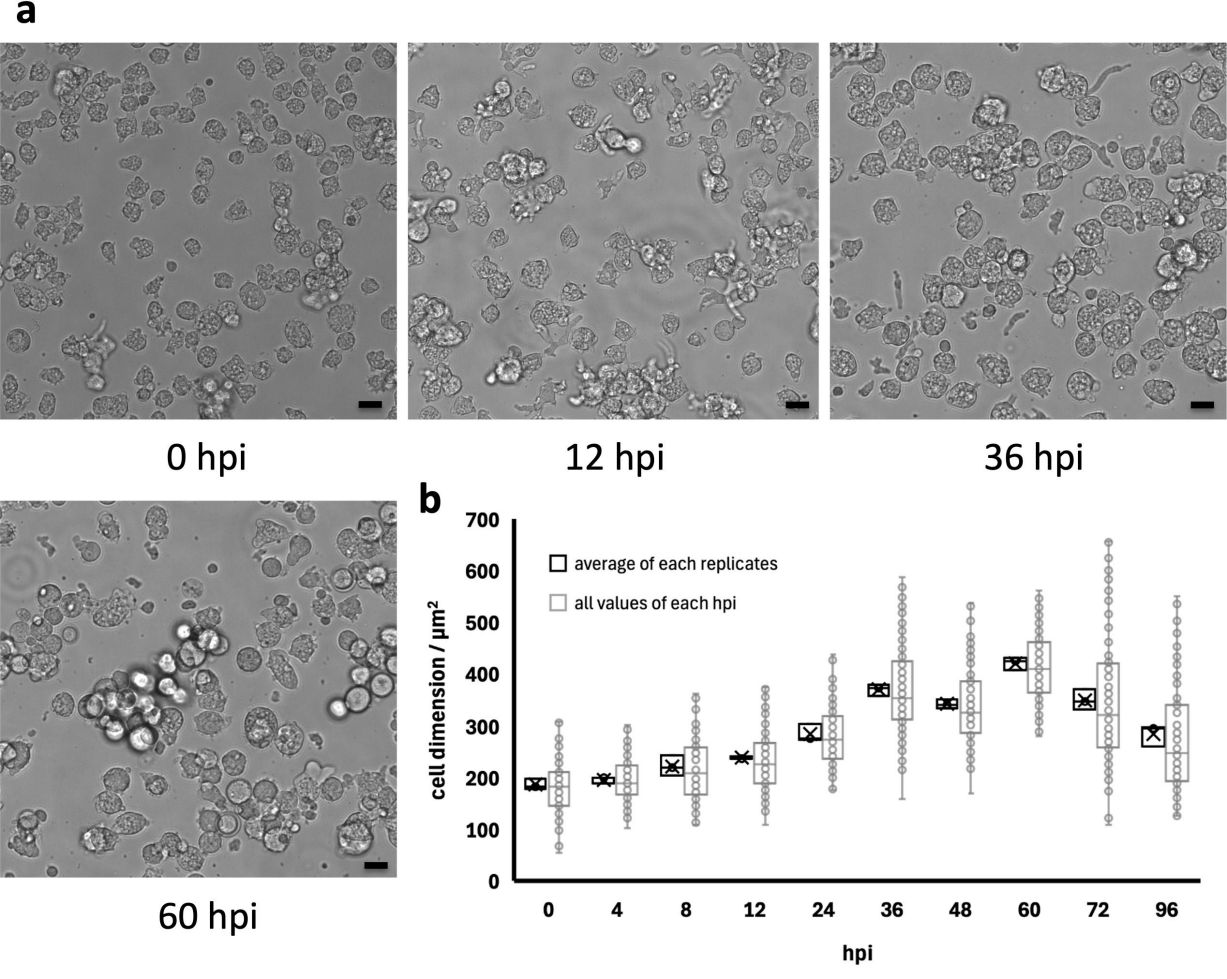
CPE of the host cell size by ushikuvirus infection. (**a**) Phase-contrast microscopic observations of vermamoeba cells after ushikuvirus infection. (**b**) Changes in cell dimensions after ushikuvirus infection. Cell dimensions were measured by selecting 50 cells randomly from each image. Two images for cells at 96 hpi were selected from each independent image. The average values (black boxes) showed an increase in cell dimensions, peaking at 60 hpi. All values were plotted (gray) on the chart (*N* = 3). X labels indicate the average values of each data set, gray boxes indicate the interquartile ranges, horizontal lines in the box indicate the medians, and whiskers indicate the minimum and maximum values, respectively. Scale bars: 20 µm.

The morphological changes in vermamoeba cells after ushikuvirus infection were observed by comparison with the endosymbiotic bacterium *Candidatus* phylum *Dependentiae* strain Noda2021, isolated from a Japanese freshwater pond in 2021 (20). As mentioned above, vermamoeba cells have two morphological forms: globular and fusiform, even in healthy conditions. However, vermamoebae cultured long term in our laboratory using PYG medium initially exhibit a typical globular shape, and the number of globular cells increased throughout the culture period (Fig. 3a). In the case of Noda2021 infection, the number of vermamoeba cells exhibiting a fusiform shape gradually increased from 3 days post-infection (dpi), and eventually, most of the cells underwent lysis (Fig. 3a). In contrast, ushikuvirus infection showed a distinct CPE on vermamoeba, with cells becoming round and some fusiform by 3 dpi, which was maintained up to 6 dpi (Fig. 3a). Cell lysis was not detected in ushikuvirus infection, suggesting that ushikuviruses are released from infected cells without cell lysis, probably by exocytosis. Cell numbers (measured using a cell counting chamber) did not change under ushikuvirus infection throughout the experiment (Fig. 3b), suggesting that ushikuvirus infection immediately inhibits cell proliferation. Vermamoeba cells increased moderately in size and gradually changed to a smooth and round shape during the early stage of ushikuvirus infection, i.e., up to 36 hours post-infection (hpi) (Fig. 4). The cells were progressively enlarged, a trend that persisted until 60 hpi, when the average cell dimensions were approximately two times larger than those of uninfected cells (0 hpi). Thereafter, the cells decreased in size or lost their typical morphology. Although the cell dimension varied, there was a general decreasing trend across the population (Fig. 4b).

### Proliferation of ushikuvirus in vermamoeba

**Fig 5:**
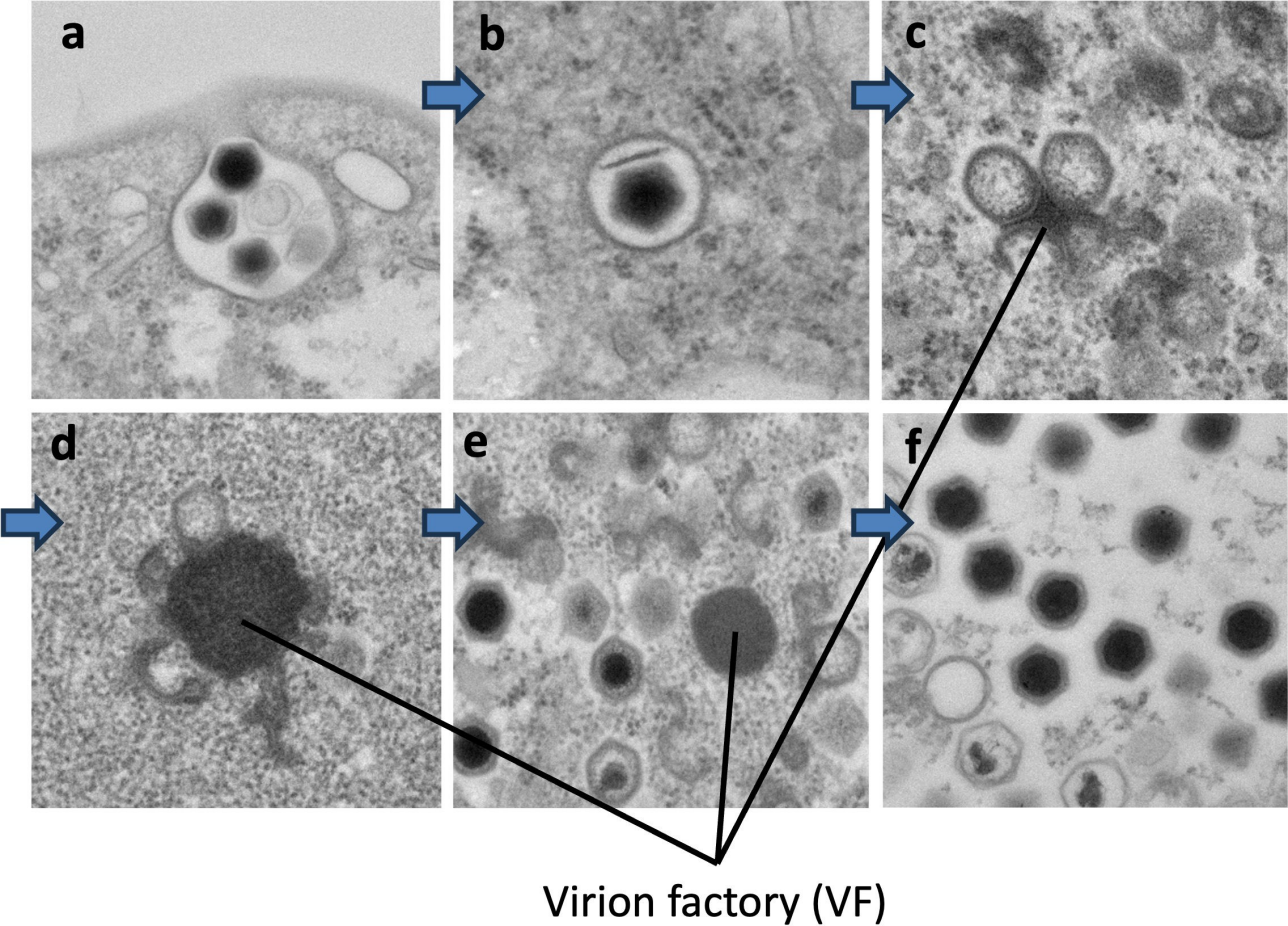
Infection and proliferation images of ushikuvirus in vermamoeba cells. The c-TEM images were acquired at 2 (**a,b**), 4 (**c,d**), 8 (**e**), and 120 (**f**) hpi. Cell infection was carried out under an unknown MOI. Viral factories are marked in the figure. The blue arrows indicate the flow of phases: from virion entry into the cell, through VF construction, and finally, to particle production.

**Fig 6:**
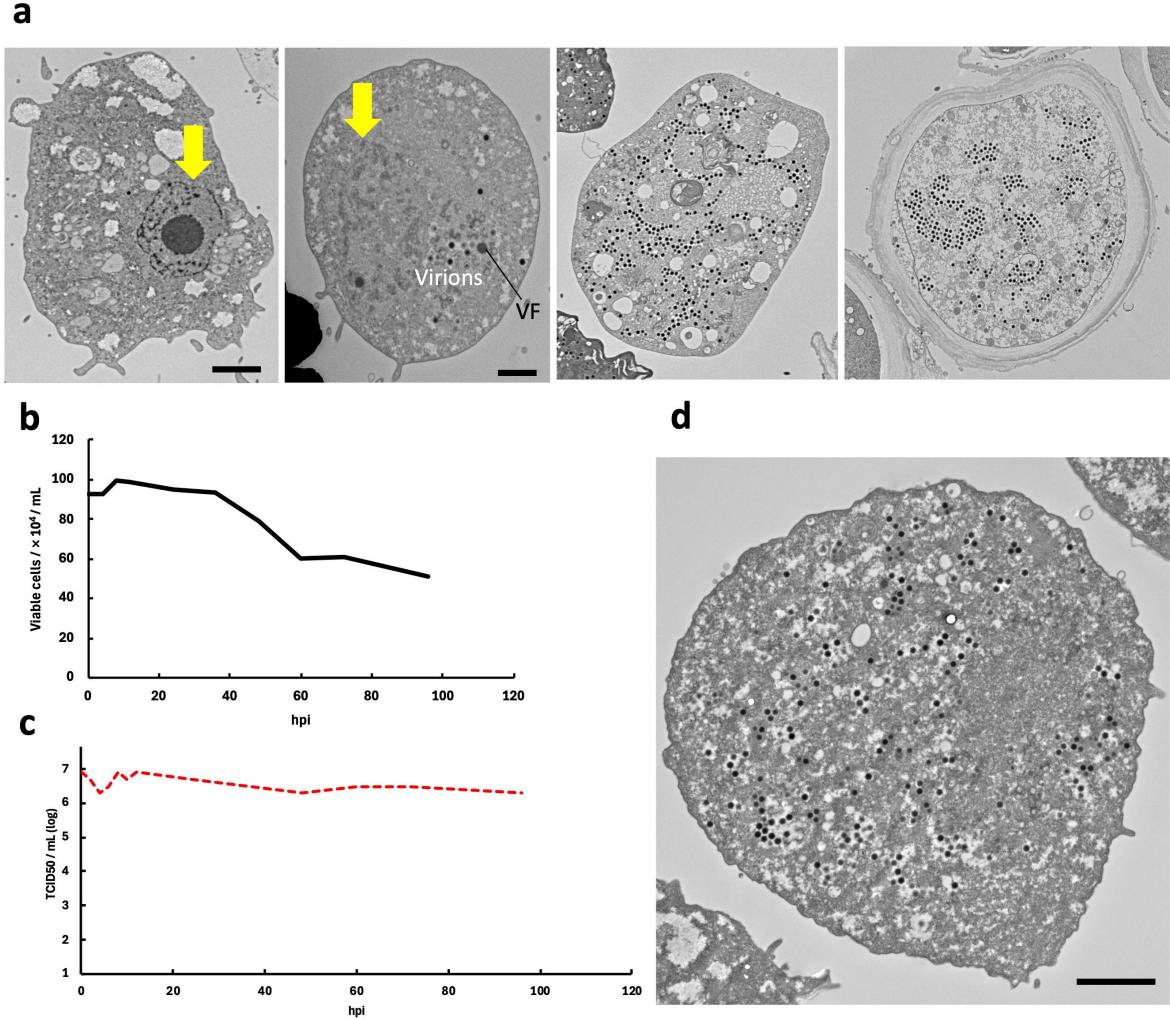
Morphological changes of ushikuvirus-infection cells. (**a**) c-TEM images of different stages of vermamoeba cells infected by ushikuvirus at 5 dpi. Yellow arrows indicate the cell nucleus (1st panel from left) and vanished nucleus (2nd panel from left). VF means virion factory. Scale bars: 2, 1, 2, and 2 µm. (**b**) Viable cells (not including small-sized rounded cells) were counted manually in the same area using a cell counter after ushikuvirus infection (MOI of 5). (**c**) The TCID_50_ of the supernatant of the ushikuvirus-infected vermamoeba cell culture was calculated at each indicated hpi (MOI of 10). (**d**) Conventional transmission electron microscopy images of vermamoeba cells infected by ushikuvirus at 60 hpi (MOI of 10). Scale bars: 2 µm.

A c-TEM observations of the ushikuvirus-infected vermamoeba cells provided clues into the infection and proliferation processes. (i) Ushikuviruses were taken up into the vermamoeba cells by endocytosis or phagocytosis (Fig. 5a). (ii) Virion uncoating occurred, followed by the formation of virion factories (VFs) within the cytoplasm of vermamoeba (Fig. 5b and c). In parallel, the nuclear membrane of virus-infected vermamoebae started to disappear (Fig. 6a). (iii) Progeny virions were produced from VFs and mature virions accumulated in the cytoplasm (Fig. 5d and e). (iv) Virion particles were finally released from the vermamoeba cells by exocytosis (Fig. 5f and 6b). As shown in Fig. 5c through e, the VFs of ushikuvirus were detected as electron-dense globular regions in the vermamoeba cytoplasm, such as the VFs of the family *Mimiviridae* (2, 24–26). Progeny virus particles are thought to form from the surface of the VFs, with initial capsid assembly followed by genomic packaging, as observed in mimivirus (Fig. 5c through e). As ushikuvirus infection progressed, the nuclear membrane of host vermamoeba cells disappeared, although vestiges of putative heterochromatin remained (Fig. 6a). This feature has also been observed in pandoravirus infection (27), but not in clandestinovirus as the host nuclear membrane does not disappear in the infection cycle (11). Furthermore, we observed some cysts containing ushikuvirus particles in the cytoplasm (Fig. 6a). The number of rounded, viable cells (cells possessing pseudopods) infected with ushikuvirus was moderately decreased at 96 hpi (Fig. 6b). The TCID_50_ (tissue culture infectious dose) value of ushikuvirus in the culture supernatant of virus-infected vermamoeba cells was maintained up to 96 hpi, despite a decrease in viable cells (Fig. 6c). These findings suggest that ushikuvirus is released by exocytosis from infected cells over time and not rapidly released from lysed cells. To confirm this, we visualized cells from a culture infected at a multiplicity of infection (MOI) of 10 using electron microscopy (Fig. 6d) and monitored their movement via time-lapse imaging (Movies S1 and S2).

### Genomic and phylogenetic characterizations of ushikuvirus

**Fig 7:**
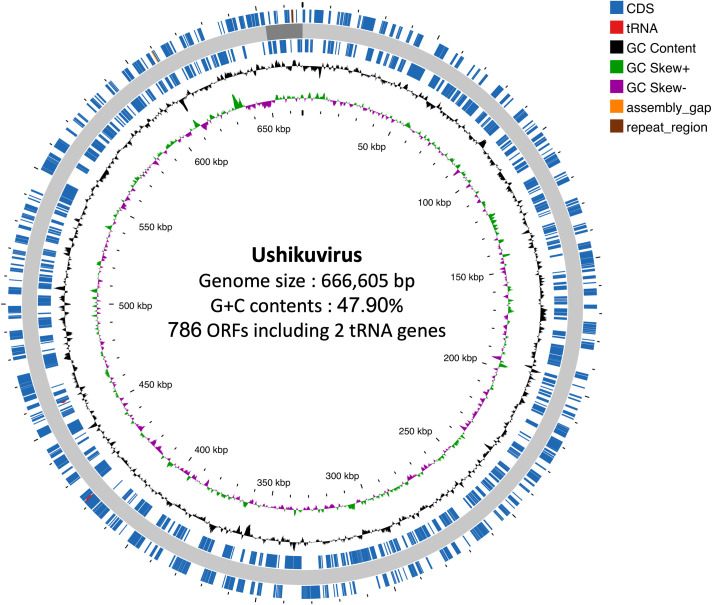
Circular representation of the ushikuvirus genome, showing from outside to inside the positive and negative strand (CDS) coding sequences (blue), including tRNA (red), GC content (black), and GC skews (green and purple).

**Fig 8:**
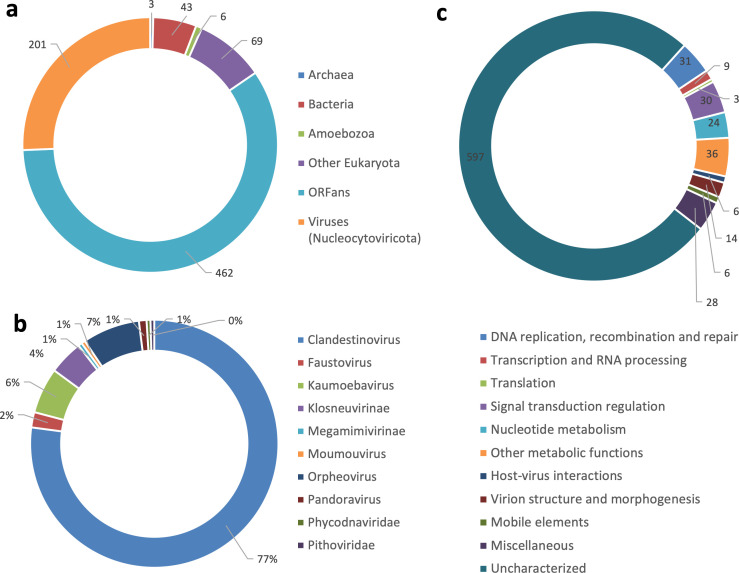
Best hits of predicted ORFs of ushikuvirus. (**a**) Pie chart showing best hits of amino acid homology between ushikuvirus and publicly available sequences of other viruses and organisms. (**b**) Pie chart showing best hits of amino acid homology between ushikuvirus and other viruses of the phylum *Nucleocytoviricota*. (**c**) Classification of ushikuvirus genes based on the functional category of genes.

**Fig 9:**
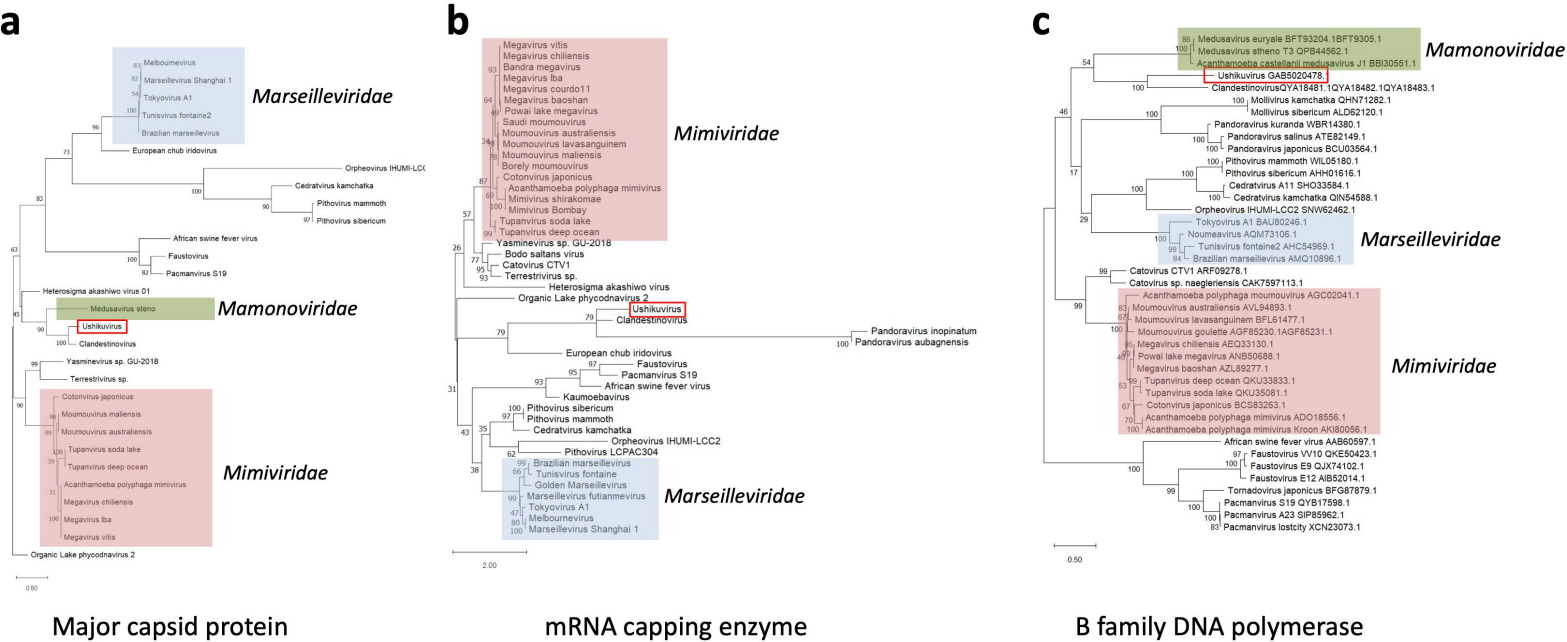
Molecular phylogenetic analysis of amino acid sequences of (**a**) MCP; (**b**) mRNA capping enzyme; (**c**) family B DNA polymerase.

**Fig 10:**
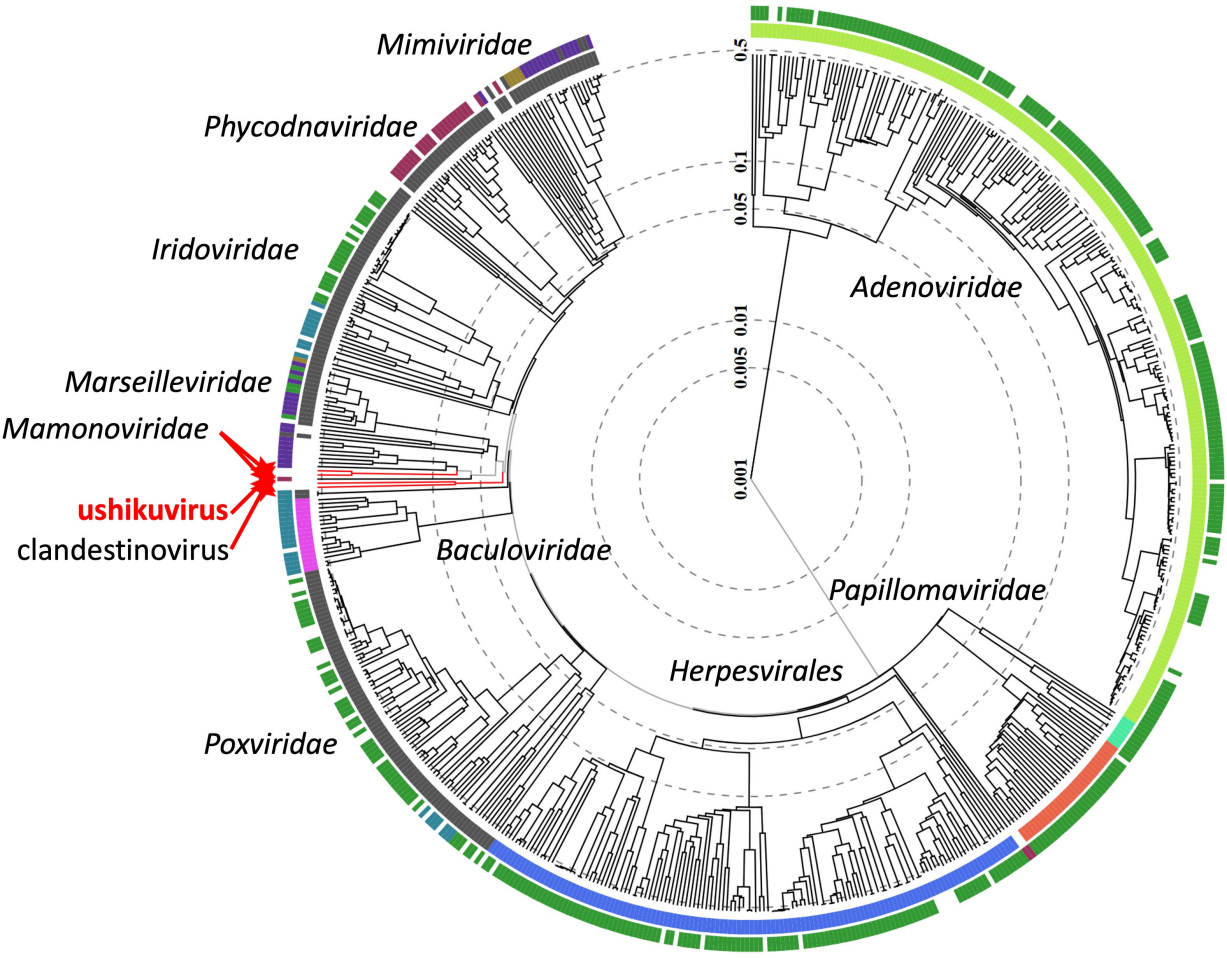
Proteomic tree based on the full-length sequences of dsDNA viruses. Each color represents the family or group of dsDNA viruses. Ushikuvirus is shown with red character and line. The clandestinovirus and family *Mamonoviridae* are also indicated using red characters and lines.

Analysis of the whole-genome sequence of ushikuvirus resulted in the reconstruction of two contigs of 652,555 and 14,050 bp, indicating that the ushikuvirus genome is at least 666,605 bp in length, has a GC content of 47.90%, and contains 784 genes (and two tRNA genes) (Fig. 7). Annotation of each open reading frame (ORF) revealed that the majority were classified as ORFans (58%), and 25% of the ORFs shared sequences similar to other viruses in the phylum *Nucleocytoviricota* (Fig. 8a). Among the ORFs with similarity to other viruses, the majority (80%) shared similarity with sequences from clandestinovirus (11) (Fig. 8b). Clandestinoviruses are closely related to the family *Mamonoviridae,* which includes the genus *Medusavirus*, but their host is different from that of medusavirus, *Acanthamoeba castellanii*. From our observations, ushikuvirus, like clandestinovirus, infects only vermamoeba and not acanthamoeba. A functional enrichment analysis of the ORF profile showed a pattern consistent with other giant viruses, and thus no specific functional genes were reported for ushikuvirus (Fig. 8c). Molecular phylogenetic analyses based on major capsid protein (MCP), mRNA capping enzyme, and family B DNA polymerase genes supported the close relationship between ushikuvirus and clandestinovirus (Fig. 9). The genomes of the family *Mamonoviridae*, except for newly discovered medusavirus euryale (8), encode a full set of histones (H1, H2A, H2B, H3, and H4), as observed in eukaryote genomes and clandestinovirus (6, 7, 11). The ushikuvirus genome was also found to encode a full set of histones, although the genes encoding H2A and H2B were fused together, similar to the H2A-H2B fusions of clandestinovirus and marseillevirus (Table 1). A recent study suggests that the linker histones of medusavirus do not function as nucleosome linkers (10). Thus, the linker histones of ushikuvirus and clandestinovirus may not function as “linkers.” These genomic features strongly suggest that ushikuvirus is closely related to clandestinovirus and the family *Mamonoviridae*, and these findings are further supported by proteomic trees constructed using ViPTree (Fig. 10).

**TABLE 1:** Histones encoded by viruses

	No. of histones					Notes	Total no. of coding sequences
Species (family)	H1	H2A	H2B	H3	H4		
*Medusavirus medusae*	1	1	1	1	1		5
*Medusavirus sthenus*	1	1	1	1		H3/H4 fused protein	4
*Medusavirus euryale*		1	1	1			3
Clandestinovirus	1	1		1	1	H2A/H2B fused protein	4
Ushikuvirus	1	1		1	1		4
Tokyovirus A1		1	1	1			3
Lausannevirus		1	1	1			3
Insectomime virus		1	1	1			3
Brazilian marseillevirus		1	1	1			3
Golden marseillevirus		1					1
Pandoraviruses			1				1
*Mimiviridae*							0
Mollivirus							0
*Pithoviridae*							0
Extended *Asfarviridae*							0

### Unique structure of the ushikuvirus capsid

**Fig 11:**
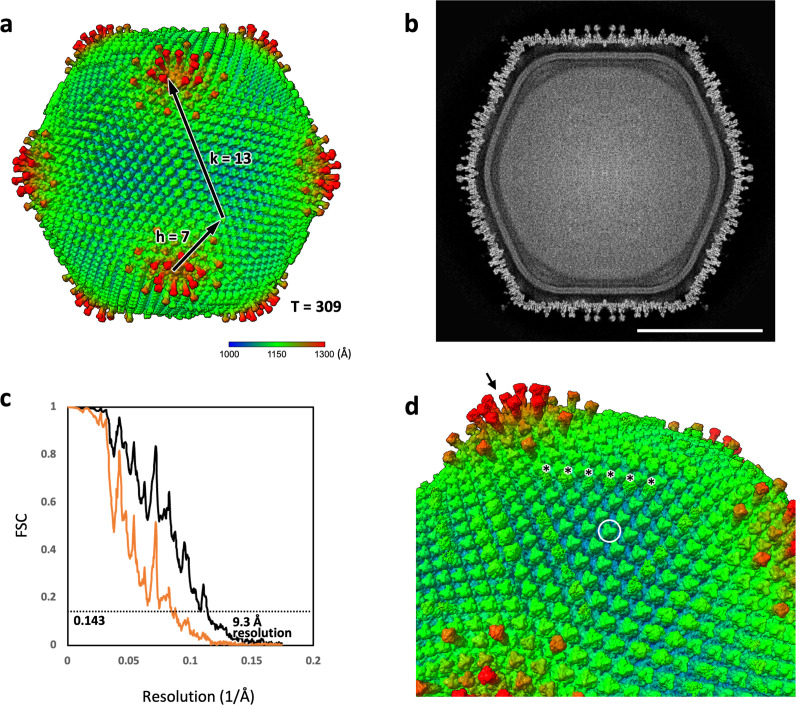
Cryo-EM SPA of the ushikuvirus particle. (**a**) Surface representation of the ushikuvirus particle at 9.3 Å resolution by imposing icosahedral symmetry, showing that the capsid is composed of T = 309 icosahedron (h = 7, k = 13). The radius distribution is shown in different colors. (**b**) Cryo-EM map of a center slice of the ushikuvirus particle. The viral DNA is surrounded by the nuclear membrane in the capsid. Scale bar represents 100 nm. (**c**) The gold-standard Fourier shell correlation (FSC) curve indicates a map resolution of 9.3 Å with a capsid mask. Unmasked FSC is presented in orange. (**d**) A magnified image of the capsid surface. The capsid surface is surrounded by multiple cap proteins (arrow and white circle) and fibers (asterisks).

**Fig 12:**
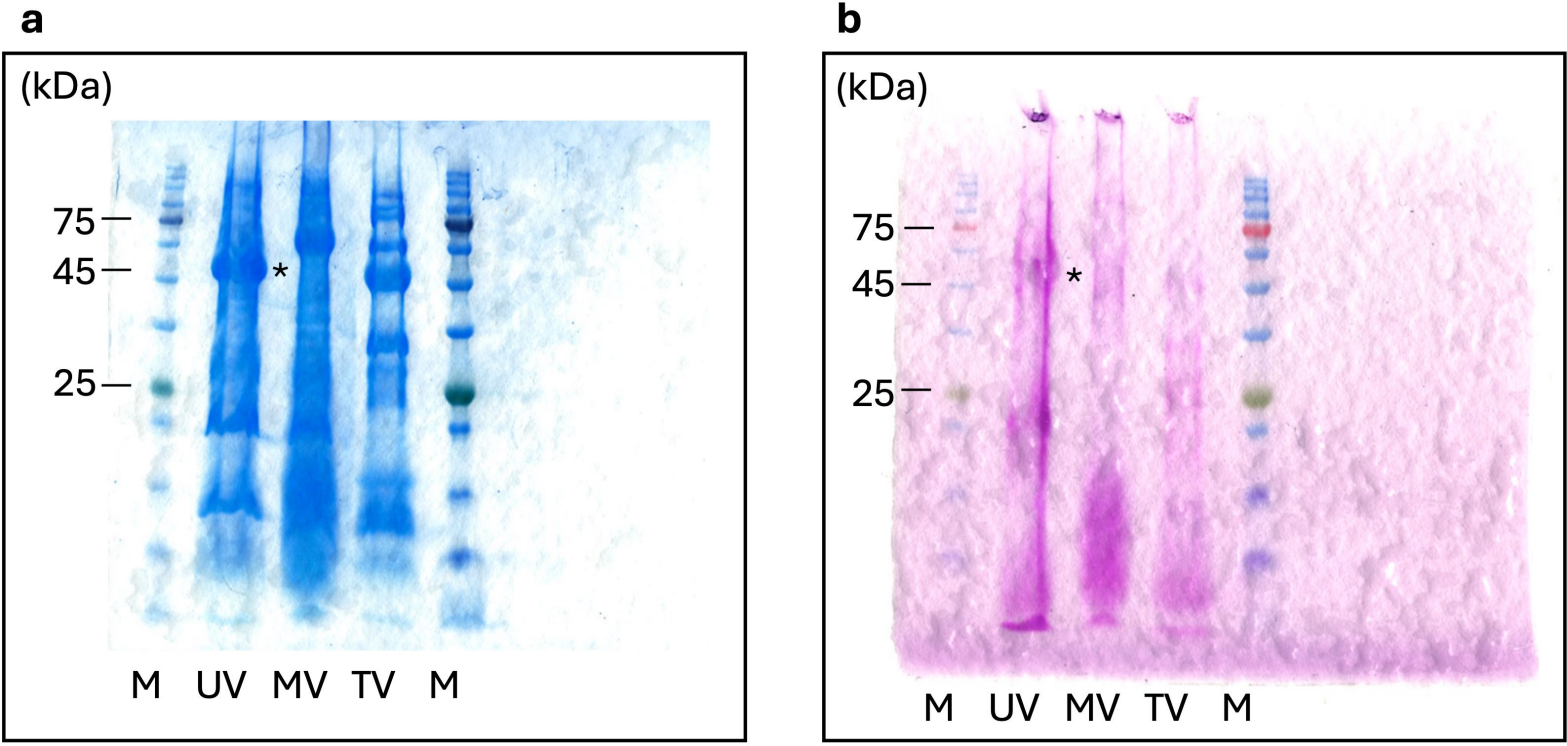
PAS staining of ushikuvirus and other viruses. Purified viral particles were subjected to SDS-PAGE, followed by (**a**) CBB and (**b**) PAS staining. Asterisks indicate putative ushikuvirus MCP in a and unknown carbohydrate-attached proteins in **b**. UV, MV, and TV indicate ushikuvirus, Mimivirus shirakomae, and tokyovirus, respectively.

As mentioned above, the morphological features of the ushikuvirus viron, determined using cryo-TEM and c-TEM analyses, were considerably similar to those of medusavirus (6), including the capsid diameter and the presence of numerous spikes on the capsid surface (Fig. 11). To elucidate the detailed structural characteristics of the viral capsid, a cryo-EM single-particle analysis (SPA) of ushikuvirus was performed. As a result, the capsid array on the viral surface was reconstructed at 9.3 Å resolution using a capsid-specific mask (Fig. 11a through c). Notably, ushikuvirus capsid with a diameter of 250 nm, except for the surface spikes, showed T = 309 icosahedron consisting of h = 7 and k = 13 (Fig. 11a), which is similar to those of *Marseilleviridae* viruses, not *Mamonoviridae* viruses with a diameter of ~260 nm showing T = 277 icosahedron consisted of h = 7 and k = 12 (6). The fact suggested that MCPs of ushikuvirus are more closely packed in the capsid than that of medusaviruses. To elucidate why the MCP of ushikuvirus is closely packed, we modeled its structure using AlphaFold2 (28) and compared it to the MCPs of tokyovirus (*Marseilleviridae*) and medusavirus (*Mamonoviridae*) (Fig. S1). The results revealed that these structures are highly similar, consisting primarily of two jelly-roll motifs. This suggests that the difference in capsid packing is attributable to the formation of a minor capsid protein (mCP) that maintains the MCP array, with ushikuvirus possessing a unique mCP structure, warranting further structural analysis in future studies. The viral DNA was surrounded by an inner membrane, similar to other giant viruses (Fig. 11b). The surface of the ushikuvirus capsid was covered with multiple spikes (Fig. 11d), with several longer spikes distributed around the fivefold vertices (arrow in Fig. 11d), similar to those of the medusavirus capsid (6, 29, 30), and the diameter of the capsid including the spikes reached approximately 270 nm (Fig. 11a and b). However, the other spikes were relatively shorter and diverse compared to those of medusavirus. The shortest spikes formed a unique “cap” structure (white circle in Fig. 11d) that was not observed in medusavirus. Among these, sixteen of the capsomeres arranged in a straight line of six each around the threefold axis were particularly decorated with the small amounts of fibrous structures on their surfaces (asterisks in Fig. 11d). To examine whether the capsid surface, including the flexible fibrous structures, is composed or arranged with glycans, periodic acid-Schiff (PAS) staining was performed using ushikuvirus proteins. The result suggested that the capsid of ushikuvirus may be decorated with some glycans attached to the capsid proteins with a molecular weight slightly larger than that of MCP (Fig. 12). Data shown in Fig. 12a and b indicate that Mimivirus shirakomae and tokyovirus have PAS signals, consistent with previous reports showing that mimivirus has surface fibrils containing glycoprotein and tokyovirus has carbohydrate chains on its surface (21, 24, 31–33).

### Putative role of GMC-oxidoreductase

**Fig 13:**
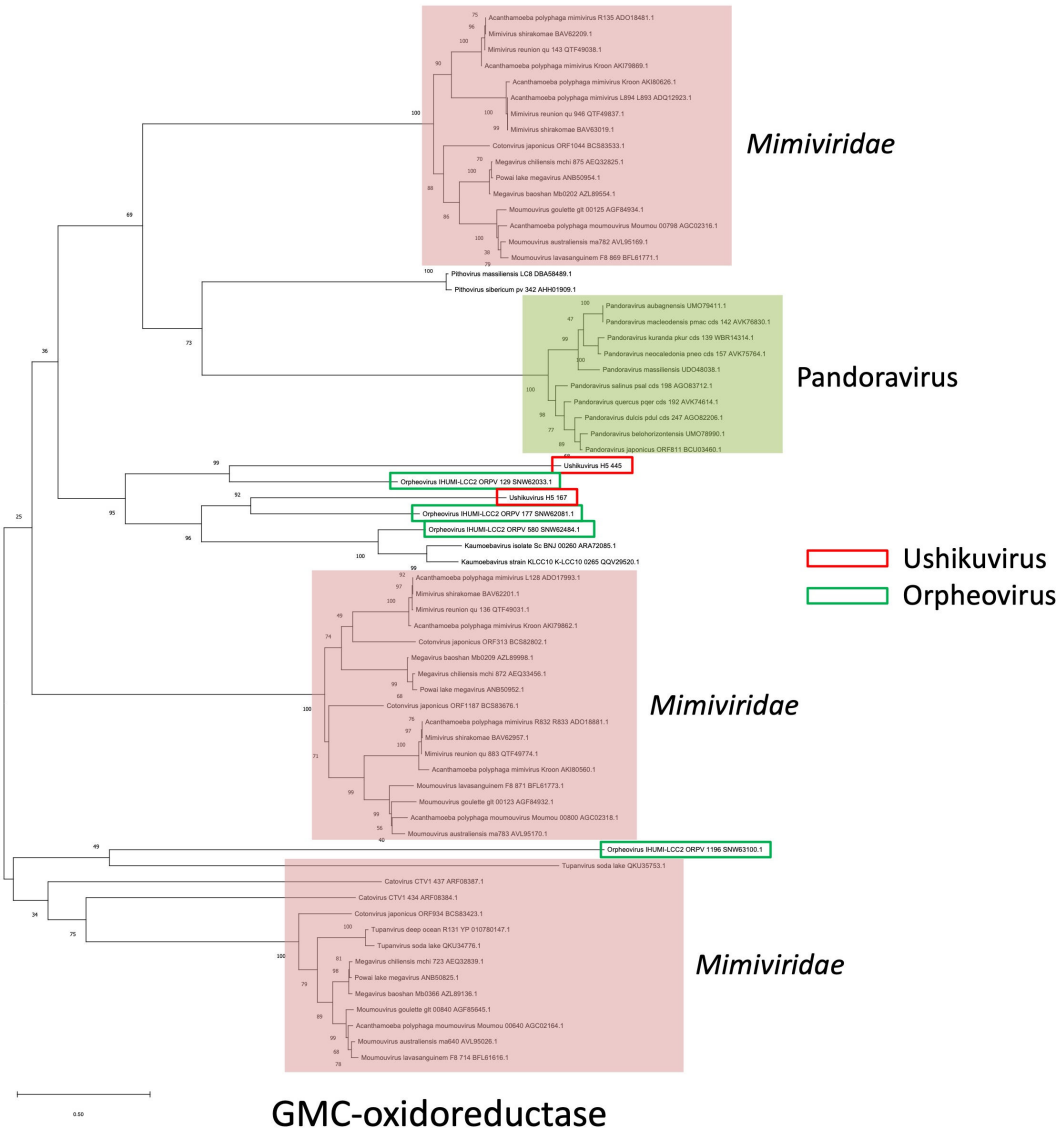
Molecular phylogenetic analysis of amino acid sequences of GMC-oxidoreductase. Ushikuvirus and orpheovirus are indicated by red boxes.

**Fig 14:**
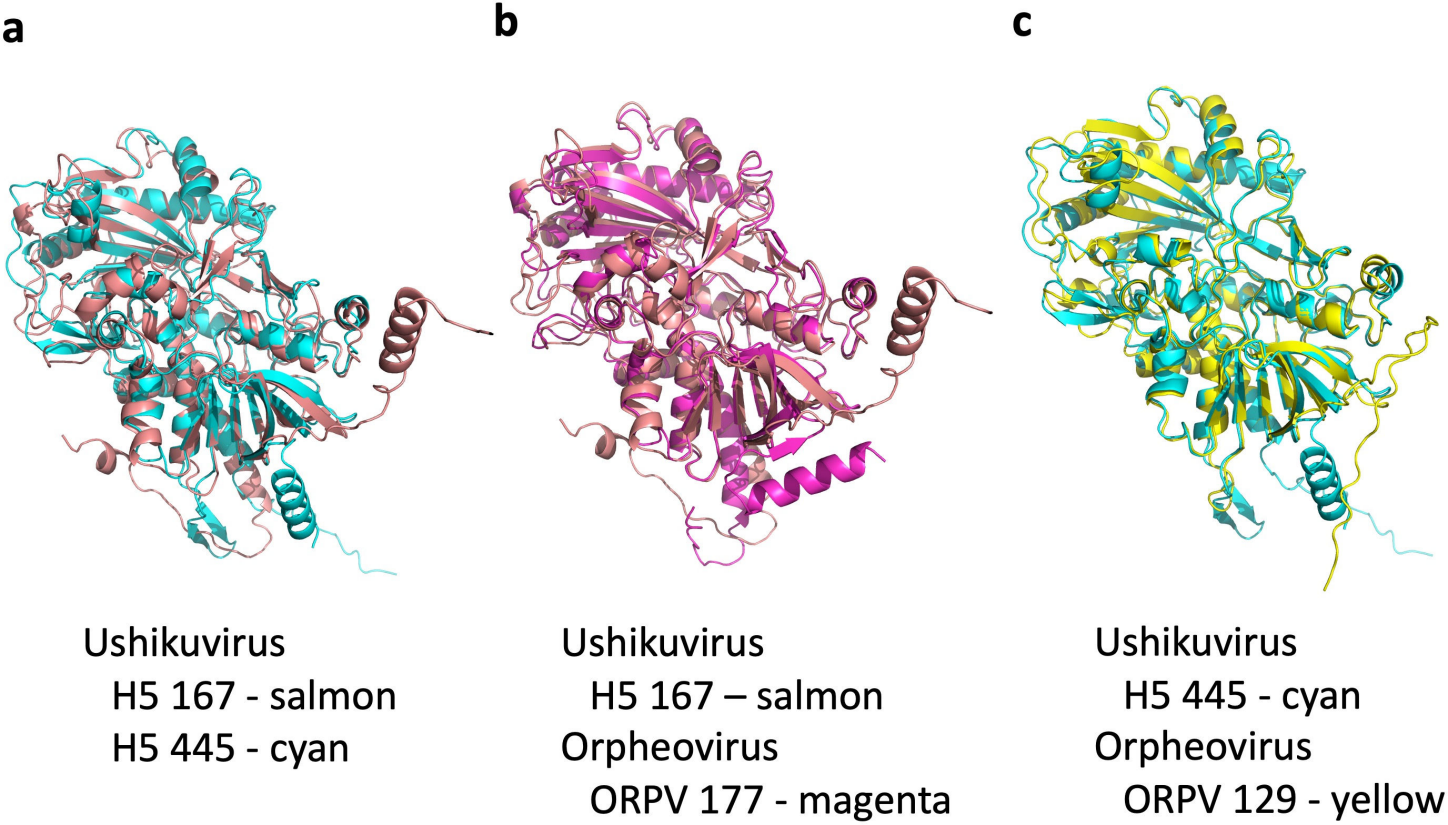
Predicted tertiary structures of GMC-oxidoreductases of ushikuvirus and orpheovirus. (**a**) Structural comparison of two ushikuvirus GMC-oxidoreductases. (**b**) Structural comparison of ushikuvirus H5 167 and orpheovirus ORPV 177. (**c**) Structural comparison of ushikuvirus H5 445 and orpheovirus ORPV 129.

In the family *Mimiviridae*, GMC-oxidoreductase is responsible for the structure and function of surface fibrils (24, 32, 34). Ushikuvirus harbored two GMC-oxidoreductase genes that are suggested to contribute to the formation of surface fibrils in mimiviruses and are also encoded by giant viruses possessing fibrous structures on the capsid surface, such as pandoravirus (27) and vermamoeba-infecting orpheovirus (15). A molecular phylogenetic analysis revealed that ushikuvirus GMC-oxidoreductases are closely related to orpheovirus GMC-oxidoreductases (Fig. 13). Among the three GMC-oxidoreductases encoded by the orpheovirus genome, ORPV 177 was similar to ushikuvirus GMC-oxidoreductase H5 167, and ORPV 129 was similar to ushikuvirus H5 445 (Fig. 13). Protein structure prediction using AlphaFold3 also revealed that these two “sets” of ushikuvirus-orpheovirus GMC-oxidoreductases are structurally homologous to them (Fig. 14). These proteins involved in the capsid surface fibers may be a key component to infect vermamoeba.

## DISCUSSION

Since the discovery of the first medusavirus strain, *Acanthamoeba castellanii* medusavirus (*Medusavirus medusae*), in 2019, there has been a gradual increase in the reports of members of the family *Mamonoviridae* and closely related taxa, including medusavirus stheno (*Medusavirus sthenus*) discovered from a freshwater river in Japan in 2021, medusavirus euryale (without an assigned species name) discovered from a freshwater river in South Korea in 2025, and clandestinovirus discovered in wastewater in France in 2021 (6–8, 11). Clandestinovirus is phylogenetically closely related to medusaviruses but was not classified in the family *Mamonoviridae* because its host is vermamoeba rather than acanthamoeba (5, 11). These results prompted us to evaluate the differences between *Mamonoviridae* viruses that infect acanthamoeba and clandestinovirus that infects vermamoeba. The discovery of ushikuvirus, which was closely related to clandestinovirus, in this study further encouraged us to evaluate the characteristics of these viruses that contribute to differences in host species.

Besides the host, medusavirus and ushikuvirus showed important differences in many aspects, including genome sizes, number of ORFs, and capsid surface structure. The genome of ushikuvirus is 666 kbp (Fig. 7), which is similar to the genome size of clandestinovirus (i.e., 582 kbp) (11), but different from viruses in the family *Mamonoviridae* (medusae: 381 kbp, sthenus: 362 kbp, euryale: 369 kbp) (6–8). Similarly, the number of ORFs of ushikuvirus (Fig. 8) is slightly higher than that of clandestinovirus (11). The relationship between genome size and number of ORFs and the differences in host amoeba has not been established. Alternatively, it is possible that the capsid structure of ushikuvirus (Fig. 11) is involved in the host difference between ushikuvirus and medusavirus.

Ushikuvirus exhibited a unique structure on the capsid surface (Fig. 11). Both ushikuvirus and medusavirus have numerous spike structures on the capsid surface, including regular and long spikes (22, 33). In addition, wide spikes have been identified in medusavirus (29, 30) but were not observed in ushikuvirus particles (Fig. 11). In both ushikuvirus and medusavirus, long spikes were observed around the fivefold vertices of the icosahedral capsid (Fig. 11) (29, 30). Interestingly, the MCPs of ushikuvirus have unique “cap” structures (Fig. 11) that are not observed in medusavirus particles (29, 30). Cryo-EM SPA of ushikuvirus suggested that all MCPs of ushikuvirus have “cap” structures, while some of them possess additional fibrous structures on the top (Fig. 11). The most studied fibrous structure on the giant virus capsid is the surface fibrils of mimivirus, which is thought to be involved in viral attachment to the surface of acanthamoeba cells. A study of a mutated mimivirus (M4 strain) lacking surface fibrils revealed several genes involved in surface fibril structure and function, including the GMC-oxidoreductase gene (32, 35). It was suggested that the GMC-oxidoreductase functions in the assembly of the surface fibrils in mimiviruses through an unknown mechanism (24). In addition, orpheovirus has thin fibrous structures on the capsid, and its genome is known to encode GMC-oxidoreductase (15). The ushikuvirus genome also contains a GMC-oxidoreductase gene, which is similar to the corresponding gene in orpheovirus, suggesting that, as in mimiviruses, there is a relationship between the fibrous structure of ushikuvirus particles and ushikuvirus GMC-oxidoreductase (Fig. 13 and 14). During cell imaging, we were unable to obtain data on the genome packaging mechanism of ushikuvirus; therefore, the localization of this protein to the genome fiber—a phenomenon observed during mimivirus genome packaging (34, 36)—was not detected. It has been reported that CPE in orpheovirus-infected vermamoeba cells shows a fusiform shape (37) that is different from CPE in ushikuvirus-infected vermamoeba (Fig. 3a). Furthermore, as shown by the PAS staining results (Fig. 12), if the fibrous structure of ushikuvirus contains glycans, this may be involved in the unique infection characteristics, leading to the slightly enlarged cells (Fig. 4). In conclusion, the fibrous structure of ushikuvirus might significantly affect its infection cycle. Compared to medusavirus and clandestinovirus, a distinctive characteristic of ushikuvirus is its long infection cycle, exhibiting a notably slower rate of CPE induction than its close relatives. We hypothesize that this surface structure causes a deceleration in the infection rate; this scenario is supported by observations within the family *Mimiviridae,* where the absence of viral surface fibers in infection kinetics results in a faster overall rate of viral replication and induction of CPE (38).

**Fig 15:**
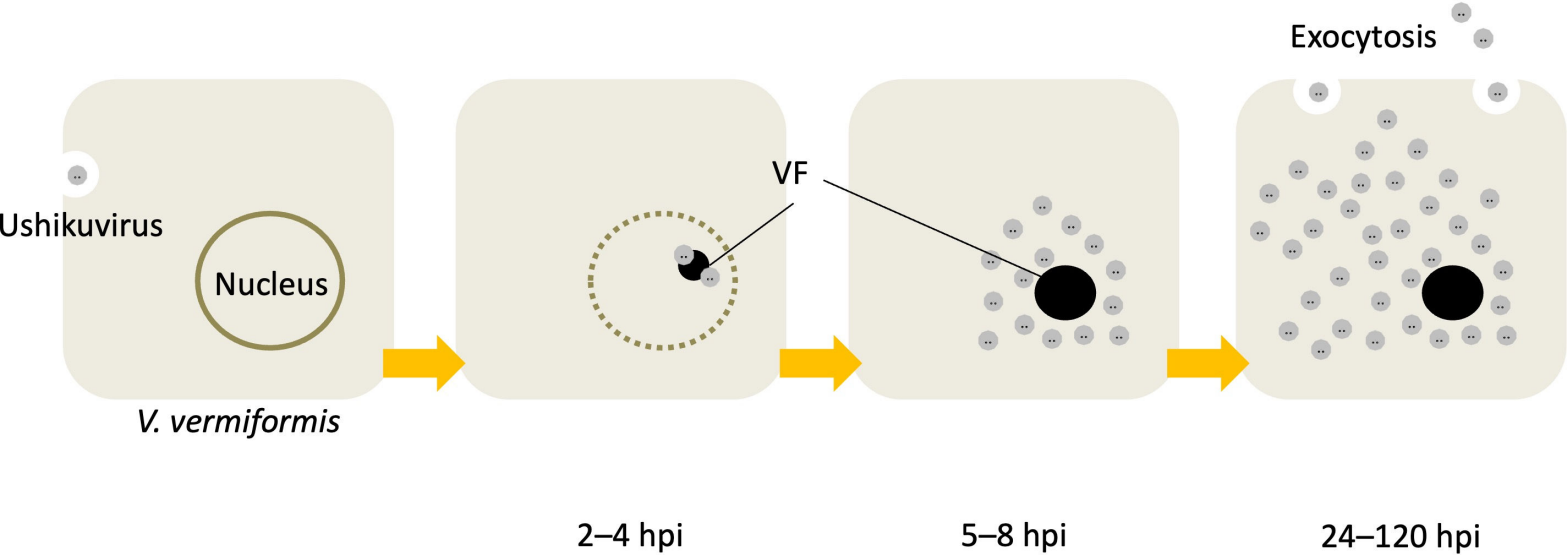
A schematic model of ushikuvirus proliferation and release. The temporal reference shown indicates the estimated time for each stage.

Notably, giant viruses infecting acanthamoeba (e.g., mimivirus, marseillevirus, medusavirus, and pandoravirus) do not induce cell enlargement when they infect healthy cells. Instead, virus infection induces cell compaction, and this change occurs within 24 hpi (39). In contrast, cells infected with mimivirus and moumouvirus belonging to the family *Mimiviridae* shrink to less than 70% of their original size at 6 hpi, whereas cells infected with megaviruses show no change in dimensions (40). On the other hand, cells infected with ushikuvirus enlarged to approximately twice the dimensions of uninfected cells on average, with some individual cells increasing to more than seven times their original size. The curve of cell dimensions (Fig. 4b) suggested that the duration of the replication cycle of ushikuvirus infection was approximately 60 hpi or more. This is consistent with the finding that vermamoeba-infected giant viruses have a longer multiplication cycle than giant viruses infected with acanthamoeba (37). However, we assessed the replication cycle under CPE without viral titer measurements at each infection time point (Fig. 6c). Although we did not measure the volume of infected cells, ushikuvirus-infected cells may have become flatter than non-infected cells, resulting in enlargement of cell dimensions when viewed from above. In that case, it can be hypothesized that the cell flattening is caused by increasing attachment of cells to flasks via the release of ushikuvirus particles with fibrous structures containing glycans (Fig. 11 and 12). It is believed that ushikuvirus particles are gradually released by exocytosis from infected vermamoeba cells without cell lysis, which promote cell flattening. Another possible scenario for cell enlargement is that ushikuvirus particles have not yet been released from the cells, leading to their accumulation in the cytoplasm of infected cells, causing cell swelling. A hypothetical model for the mechanism of ushikuvirus proliferation and release is schematically shown in Fig. 15. It is also possible that in an aqueous environment, swollen, floating cells may excrete virions over a wide area for a long period of time.

While the replication and proliferation strategy of ushikuviruses is similar to that of members of the family *Mamonoviridae*, notable differences are also observed. However, the possible evolutionary scenario is unclear because the molecular phylogenies of ushikuvirus' core proteins have not been elucidated. For example, sequence similarity in MCP and B family DNA polymerases was detected between ushikuvirus and clandestinovirus and their sister clade, medusaviruses (Fig. 9a and c), but both ushikuvirus and clandestinovirus genomes encode mRNA capping enzymes that are not encoded by medusaviruses of the family *Mamonoviridae* (Fig. 9b). The proteomic tree of DNA viruses suggests that the genomic evolution of ushikuvirus and clandestinovirus is earlier than the diversification of the family *Mamonoviridae* (Fig. 10), prompting us to hypothesize that the divergence of the ancestors of ushikuvirus and clandestinovirus from the ancestor of the family *Mamonoviridae* coincides with the diversification of the class *Discosea,* which includes acanthamoeba*,* and the class *Tubulinea,* which includes vermamoeba. Regarding the effects of these viruses on the host nucleus, interesting evolutionary scenarios are possible since medusaviruses of the family *Mamonoviridae* and clandestinovirus do not disrupt the nuclear membranes and replicate within the host cell nucleus, while ushikuvirus disrupts the host nuclear membrane (Fig. 6). In pandoravirus, whose particle size is significantly larger than that of other giant viruses, loss of nuclear membranes has been observed following virus infection (27). Although it is not clear why the disassembly of the nucleus occurs upon infection with these viruses, the genomes of medusavirus do not encode RNA polymerase, mRNA capping enzyme, or DNA topoisomerase II, resulting in a strong dependency on the host cell nucleus, which they then use as a “virion factory” (6, 9). Ushikuvirus does not need to use host enzymes because the virus itself encodes these proteins and therefore may not need to persist in the host cell nucleus. Moreover, we observed cyst formation in the ushikuvirus-infected culture environment. Encystment of infected cells has been uniquely reported for faustovirus mariensis (41), a virus infecting vermamoeba, and is a common feature among genus *Medusavirus* species infecting acanthamoeba (6). However, a unique characteristic of these viruses is their ability to induce encystment in almost all cells in the culture environment. Specifically, *F. mariensis* induced encystment in most cells in the culture environment at an MOI exceeding 1. However, similar observations have not yet been reported in the ushikuvirus-infected environment, necessitating further studies.

Several giant viruses infecting vermamoeba have been isolated, including ushikuvirus, clandestinovirus, faustovirus, fadolivirus, orpheovirus, kaumoebavirus, yasminevius, and tupanvirus (11–18). Tupanvirus, in particular, behaves as “dual-acting” viruses, capable of infecting both acanthamoeba and vermamoeba (16). Tupanvirus exploits both classes of amoebae via shared features or via a set of genes for infection targeting both classes. However, ushikuvirus and most vermamoeba-infecting viruses appear to have only one set of genes for infection targeting the class *Tubulinea*. Nevertheless, the molecular similarities between ushikuvirus and the family *Mamonoviridae* indicate that the basic infection mechanism is shared between these taxa, and differences in infection strategies, including the capping of capsid proteins, addition of fibrous structures, mechanisms of nuclear membrane disassembly, and moderate release of particles using exocytosis, likely arose during ushikuvirus evolution. Taken together, this newly isolated relative of the family *Mamonoviridae* and clandestinovirus may provide key insights into virus-host interactions and the evolution of this virus group.

## MATERIALS AND METHODS

### Culture of vermamoeba, virus isolation, and virus cloning

*Vermamoeba vermiformis* was purchased from American Type Culture Collection (ATCC) as *Hartmannella vermiformis* Page strain CDC-19 (ATCC 50237). Vermamoeba cells were cultured in proteose peptone-yeast extract-glucose (PYG) medium at 26°C, similar to the conditions for acanthamoeba described previously (6, 21, 42). Water samples (50 mL) were collected from a freshwater pond, Ushiku-numa, in Ibaraki Prefecture of Japan and then stored at 4°C until inoculation to vermamoeba cells. A portion of the sample (4.5 mL) was filtered using Whatman filter paper 43, followed by filtration using a 1.2 µm syringe filter. Samples were then mixed with 2 × PYG medium (4.5 mL) and an antibiotic solution (360 µL), as described previously (6, 21, 42). The mixed solution containing the vermamoeba suspension (50 µL) was inoculated into a 96-well microplate and incubated at 26°C. After 4 days, CPE in vermamoeba cells was observed in only one well in a 96-well microplate. The supernatant of this well was added to fresh vermamoeba cells, and putative viruses were cloned by serial dilution, as described previously (6, 21, 42). The finally isolated virus was named “ushikuvirus,” reflecting the name of the freshwater pond “Ushiku-numa” where it was collected.

### c-TEM analyses

Vermamoeba cells were infected with ushikuvirus at an unknown multiplicity of infection (MOI) and incubated at 26°C. After 5 dpi, cells were collected by centrifugation at 500 × *g* for 5 min, washed with PBS, and fixed with 2% glutaraldehyde (GA) as described previously (6, 21, 42). The fixed cells were washed again with PBS, stained with 2% osmium tetroxide, and dehydrated with increasing ethanol concentrations and propylene oxide, as described previously (6, 21, 42). Dehydrated cells were embedded in EPON-812 resin (TAAB Laboratory Equipment, Aldermaston, UK), sectioned, and then visualized using a transmission electron microscope (H-7600; Hitachi, Tokyo, Japan). The c-TEM analysis was performed at Hanaichi UltraStructural Research Institute (Okazaki, Aichi, Japan).

### Cryo-EM and single-particle analysis

After 5 dpi, ushikuvirus particles released from infected vermamoeba cells were collected from the supernatant by centrifugation at 8,000 × *g* for 35 min, 4°C. After washing with PBS three times, virions were resuspended with PBS. Then, 2 µL of the virus suspension was applied to QuantiFoil R1.2/1.3 copper grids (QuantiFoil GmbH, Thuringia, Germany) and plunge-frozen using a Vitrobot Mk. IV (Thermo Fisher Scientific: TFS). Cryo-TEM imaging and grid screening for SPA were performed on a 200kV JEM-2200FS electron microscope (JEOL) using a 626 cryo-specimen holder (Gatan). For SPA, a 300 kV Titan Krios G4 electron microscope (TFS) equipped with a C-FEG electron source, Falcon 4i direct detector, and Selectris-X energy filter. Micrographs were acquired using Tomography 5 software rather than EPU software both implemented on the microscope as the high contrast of the viruses caused the hole finding and centering algorithm of EPU to misidentify most holes and skip acquisitions as a result. Micrographs were acquired at 64,000× (effective magnification 1.912 Å/pixel) with a total dose of 8 e^-^/Å^2^. Micrograph movies were imported into RELION 5 and motion-corrected using the RELION (43–46) implementation of the MotionCor2 algorithm (47), before contrast transfer function (CTF) estimation with CTFFIND 4 (version 4.1.14) (48). Micrographs with a poor CTF fit were removed manually, and 79 particles were picked across 40 micrographs. These particles were extracted into 1,600 pixel boxes (downsampled to 160 pixels) with an effective pixel size of 19.12 Å/pixel. 2D classification into five classes was performed, and 54 virus particles were selected in one class for template-based autopicking. This resulted in 8,598 automatically picked particles, which were extracted into 1,536 pixel boxes (downsampled to 192 pixels) and 2D-classified into 40 classes with a mask diameter of 2,650 Å using the expectation/maximization algorithm. Two rounds of 2D classification were performed, with a total of 1,600 particles finally selected. An initial model was generated with no symmetry imposition and aligned to I1 symmetry, which the selected particles were classified and refined against in 3D. Particles were extracted into 1,536 pixel boxes (downsampled to 384 pixel boxes, effective pixel size 7.648 Å/pixel), 3D-classified, and re-extracted into 1,536 pixel boxes (downsampled to 768 pixel boxes, effective pixel size 3.824 Å/pixel), resulting in a 10 Å resolution reconstruction (Fig. S2). CTF refinement was carried out (defocus and astigmatism refinement, followed by beam tilt estimation), followed by further 3D refinement. Finally, particles were re-extracted into 1,536 pixel boxes (downsampled to 1,024 pixel boxes, effective pixel size 2.868 Å/pixel), and a last round of 3D refinement was carried out. Ewald sphere correction (45) was performed on the half-sets, and post-processing resulted in a final resolution of 9.3 Å with a capsid mask (Fig. 11c; Fig. S2).

### Infection cycle analysis

Vermamoeba cells were cultured in PYG medium in a 25 cm^2^ culture flask and exposed to ushikuvirus particles. Images of infected and non-infected cells at 0, 1, 2, 3, 4, 5, and 6 dpi were captured using an all-in-one fluorescence microscope (BZ-X800/X810, Keyence Co., Tokyo, Japan) with a 20× objective lens. For comparison, the vermamoeba-infecting *Dependentiae* strain Noda2021 (20) was independently used to evaluate the culture cells. Images of infected vermamoeba cells were captured as described above. Furthermore, c-TEM images of ushikuvirus-infected vermamoeba cells at 2, 4, 8, and 120 h post-infection (hpi) were obtained as described above. Independently, ushikuvirus was inoculated to vermamoeba cells at an MOI of 10. The supernatants of infected cells were collected at different time points and centrifuged at 400 × *g* for 5 min. The supernatants were titrated in 96-well plates to 5 × 10^5^ vermamoeba cells using the TCID_50_ calculator (49).

### Cell counts and dimensions

Vermamoeba cells were infected by ushikuvirus at an MOI of 5 in a 25 cm^2^ culture flask. Ushikuvirus-infected cells at each time point were pipetted and quantified using a disposable hemocytometer (WAKENBTECH Co. Ltd., Tokyo, Japan). Simultaneously, infected cells were observed and imaged using an all-in-one fluorescence microscope (Keyence Co.). ImageJ software v0.5.8 (https://imagej.net/ij/index.html) was used to measure the dimensions of 50 cells in each image. The cells were selected randomly, and independent triplicate processes were performed for image acquisition at each time point. The infected cells at 96 hpi no longer maintained their form, and thus two images were analyzed for each replicate. The measurements were performed manually.

### Genome sequencing, assembly, gene prediction, phylogenetic analysis, and AlphaFold3 analysis

Genomic DNA of ushikuvirus was prepared from viral particles using NucleoSpin tissue XS (Macherey-Nagel GmbH and Co. KG, Duren, Germany) and sequenced on the Illumina NovaSeq 6000 platform using the TrueSeq Nano DNA Library Kit. Reads were assembled *de novo* using SPAdes 3.15.05 (50) and a 652,555 bp genomic sequence was generated. Gene prediction and annotation were performed using BLAST (51). These analyses were performed by Macrogen Japan (Koto-ku, Tokyo, Japan). Finally, gene prediction and annotations were all checked by the authors using BLASTp. Gene mapping was performed using the Proksee server (https://proksee.ca, accessed on 5 December 2024). The protein sequences of family B DNA polymerase, major capsid protein, mRNA capping enzyme, and GMC-oxidoreductase of the class *Megaviricetes*, phylum *Nucleocytoviricota* were used for a phylogenetic analysis. The accession IDs of these viruses are listed in Tables S1 and S2. Sequence alignments were generated using the Muscle program in MEGA XI (major capsid protein, mRNA capping enzyme, and GMC-oxidoreductase) and MEGA 12 (family B DNA polymerase) (52). From this alignment, a maximum likelihood phylogenetic tree was constructed using MEGA XI or MEGA 12 with 1,000 bootstrapping iterations (substitution model: MCP; LG + G, mRNA capping enzyme; WAG + G, GMC-oxidoreductase; LG + G + I, family B DNA polymerase; LG + G + I). The structures of GMC-oxidoreductases of ushikuvirus and orpheovirus were estimated using AlphaFold3 (53). Different structures estimated using AlphaFold3 were aligned using PyMOL (v3.1.3: https://pymol.org/support.html).

### Glycoprotein stain

For carbohydrate chain or glycoprotein detection on the ushikuvirus capsid, a staining analysis of viral particles using Schiff’s reagents was performed. Purified ushikuvirus, mimivirus shirakomae (31), and tokyovirus (21) were subjected to SDS-PAGE (CN-15L, ATTO) with a molecular marker (WSE-7020, ATTO), followed by staining using the GlycoGel Stain Kit (Polysciences, Inc., Warrington, PA, USA) as per the manufacturer’s protocol.

### Proteomic tree

The proteomic tree was constructed using the ViPTree server (version 1.9) (54). This analysis was based on the DB and the manual addition of the following viral sequences: *Medusavirus medusae* (AP018495.1), *Medusavirus sthenus* (MW018138.1), clandestinovirus (MZ420154.1), and ushikuvirus (BAAHMQ010000001.1).

## Data Availability

The sequence data for ushikuvirus are available in GenBank (contig1:BAAHMQ010000001.1; contig2:BAAHMQ010000002.1) under BioProject PRJDB19715 and BioSample SAMD00853309, and raw reads were obtained from the Sequence Read Archive (DRS429699). The final cryo-EM map, half maps, and FSC have been deposited in the electron microscopy databank (EMDB) with the following accession code: EMD-64895.

## AFTER EPUB

*[This article was published on 24 November 2025 with text missing from Materials and Methods. The text was updated in the current version, posted on 3 December 2025.]*

## SUPPLEMENTAL MATERIAL

Supplemental material jvi.01206-25-s0001.docx Tables S1 and S2, Fig. S1 and S2, and legends for Movies S1 and S2.

Movie S1 jvi.01206-25-s0002.mp4 Four-hour time-lapse of ushikuvirus-infected cells at 58-62 hpi (MOI of 10). The time-lapse movie was created at 15 frames per second from images captured every 15 s.

Movie S2 jvi.01206-25-s0003.mp4 Four-hour time-lapse of uninfected cells. The time-lapse movie was created at 15 frames per second from images captured every 15 s.

ASM does not own the copyrights to Supplemental Material that may be linked to, or accessed through, an article. The authors have granted ASM a non-exclusive, world-wide license to publish the Supplemental Material files. Please contact the corresponding author directly for reuse.
