# Supplementary material for: A newly isolated giant virus, ushikuvirus, is closely related to clandestinovirus and shows a unique capsid surface structure and host cell interactions
Source: J Virol. 2025 Nov 24;99(12):e01206-25. doi: 10.1128/jvi.01206-25 (PMC12724268; doi:10.1128/jvi.01206-25)
Supplement: Supplemental material — Tables S1 and S2, Fig. S1 and S2, and legends for Movies S1 and S2. [file jvi.01206-25-s0001.docx]

**Supplementary information**

**A newly isolated giant virus, ushikuvirus, is closely related to clandestinovirus and shows a unique capsid surface structure and host cell interactions**

Jiwan Bae^a^, Narumi Hatori^a^, Raymond N. Burton-Smith^b,c,d^, Kazuyoshi Murata^b,c,d^, Masaharu Takemura^a^#

^a^Department of Mathematics and Science Education, Graduate School of Science, Tokyo University of Science, Kagurazaka 1-3, Shinjuku, Tokyo 162-8601, Japan

^b^Exploratory Research Center on Life and Living Systems, National Institute of Natural Sciences, 38 Nishigonaka, Myodaiji, Okazaki, Aichi 444-8585, Japan

^c^National Institute for Physiological Sciences, National Institutes of Natural Sciences, 38 Nishigonaka, Myodaiji, Okazaki, Aichi 444-8585, Japan.

^d^Department of Physiological Sciences, The Graduate University for Advanced Studies (SOKENDAI), 38 Nishigonaka, Myodaiji, Okazaki, Aichi 444-8585, Japan

Running Head: Characterization of a giant virus, ushikuvirus

#Address correspondence to Masaharu Takemura, [giantvirus@rs.tus.ac.jp](mailto:giantvirus@rs.tus.ac.jp)

**Table S1.** Accession no. of MCPs used for molecular phylogenetic analysis in Fig. 9a.

| Virus | Accession No. | Virus | Accession No. |
| --- | --- | --- | --- |
| Tunisvirus fontaine2 | KF437375.1 | Tornadovirus | BFG87830.1 |
| Acanthamoeba polyphaga mimivirus | AVG46195.1 | Moumouvirus australiensis | YP_010789708.1 |
| Megavirus chiliensis | YP_004894515.1 | Terrestrivirus sp. | AYV75818.1 |
| Megavirus lba | AGD92382.1 | Cotonvirus japonicus | YP_010841729.1 |
| Tokyovirus A1 | YP_009255130.1 | Tupanvirus soda lake | YP_010782000.1 |
| Catovirus CTV1 | ARF09335.1 | Tupanvirus deep ocean | YP_010780695.1 |
| Megavirus vitis | AVL93793.1 | Yasminevirus sp. | VBB18115.1 |
| Moumouvirus maliensis | QGR53920.1 | Organic Lake phycodnavirus 2 | ADX06358.1 |
| Melbournevirus | YP_009094806.1 | Clandestinovirus | QYA18424.1 |
| Marseillevirus Shanghai 1 | AVR53056.1 | Medusavirus sthenus | YP_010801953.1 |
| Brazilian marseillevirus | YP_009238902.1 | Cedratvirus kamchatka | QIN54264.1 |
| European chub iridovirus | QCQ67796.1 | Orpheovirus IHUMI-LCC2 | YP_009449240.1 |
| Heterosigma akashiwo virus 01 | YP_009507580.1 | African swine fever virus | AAA42721.1 |
| Pithovirus mammoth | WIL05115.1 | Pacmanvirus S19 | QYB17572.1 |
| Pithovirus sibericum | YP_009001361.1 | Faustovirus | QJX73982.1 |

**Table S2.** Accession no. of mRNA capping enzymes used for molecular phylogenetic analysis in Fig. 9b.

| Virus | Accession No. | Virus | Accession No. |
| --- | --- | --- | --- |
| Clandestinovirus | QYA18707.1 | Kaumoebavirus | QQV29416.1 |
| Cedratvirus kamchatka | QIN54133.1 | African swine fever virus | QZK26801.1 |
| Tupanvirus soda lake | YP_010781873.1 | Faustovirus | SME65026.1 |
| Cotonvirus japonicus | YP_010841781.1 | Pacmanvirus S19 | QYB17642.1 |
| Heterosigma akashiwo virus | YP_009507495.1 | Melbournevirus | AIT54895.1 |
| Acanthamoeba polyphaga mimivirus | AAV50651.1 | Megavirus courdo11 | AFX92569.1 |
| Moumouvirus australiensis | YP_010789758.1 | Megavirus baoshan | YP_010788847.1 |
| Brazilian marseillevirus | YP_009238879.1 | Orpheovirus IHUMI-LCC2 | YP_009449247.1 |
| Tunisvirus fontaine2 | YP_009507001.1 | Bodo saltans virus | YP_010778709.1 |
| Catovirus CTV1 | ARF09224.1 | Pithovirus sibericum | YP_009001355.1 |
| Terrestrivirus sp. | AYV76450.1 | Tupanvirus deep ocean | YP_010780579.1 |
| Yasminevirus sp. | VBB17906.1 | Pithovirus LCPAC304 | QBK91905.1 |
| Megavirus lba | AGD92435.1 | Tupanvirus deep ocean | YP_010780579.1 |
| Tokyovirus A1 | YP_009255104.1 | Marseillevirus futianmevirus | WRK65232.1 |
| European chub iridovirus | QCQ67761.1 | Marseillevirus Shanghai 1 | AVR53030.1 |
| Pandoravirus inopinatum | YP_009120231.1 | Moumouvirus lavasanguinem | BFL61404.1 |
| Pandoravirus aubagnensis | UMO79684.1 | Golden Marseillevirus | YP_009310325.1 |
| Organic Lake phycodnavirus 2 | ADX06468.1 | Megavirus vitis | AVL93837.1 |
| Moumouvirus maliensis | QGR53966.1 | Mimivirus shirakomae | BAV62472.1 |
| Saudi moumouvirus | AQN68341.1 | Mimivirus Bombay | AMZ02827.1 |
| Bandra megavirus | AUV58459.1 | Borely moumouvirus | QID06148.1 |
| Powai lake megavirus | YP_010776374.1 | Pithovirus mammoth | WIL05109.1 |
| Megavirus chiliensis | YP_004894563.1 |  |  |


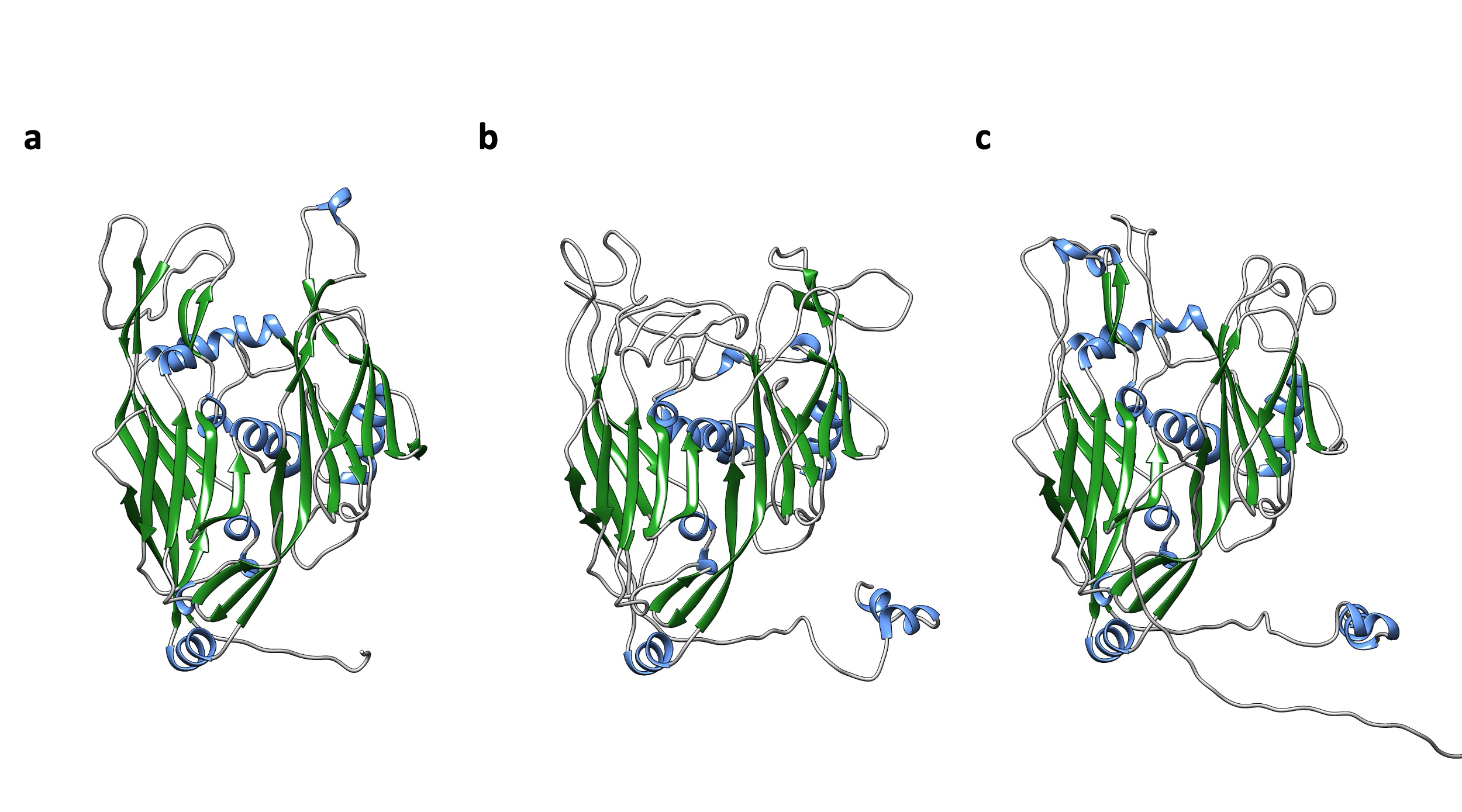


**Figure S1.** An MCP model of ushikuvirus was generated from the amino acid sequence using AlphaFold2 (a) and compared with the MCP structures of tokyovirus (family *Marseilleviridae*) (b) and medusavirus (family *Mamonoviridae*) (c).


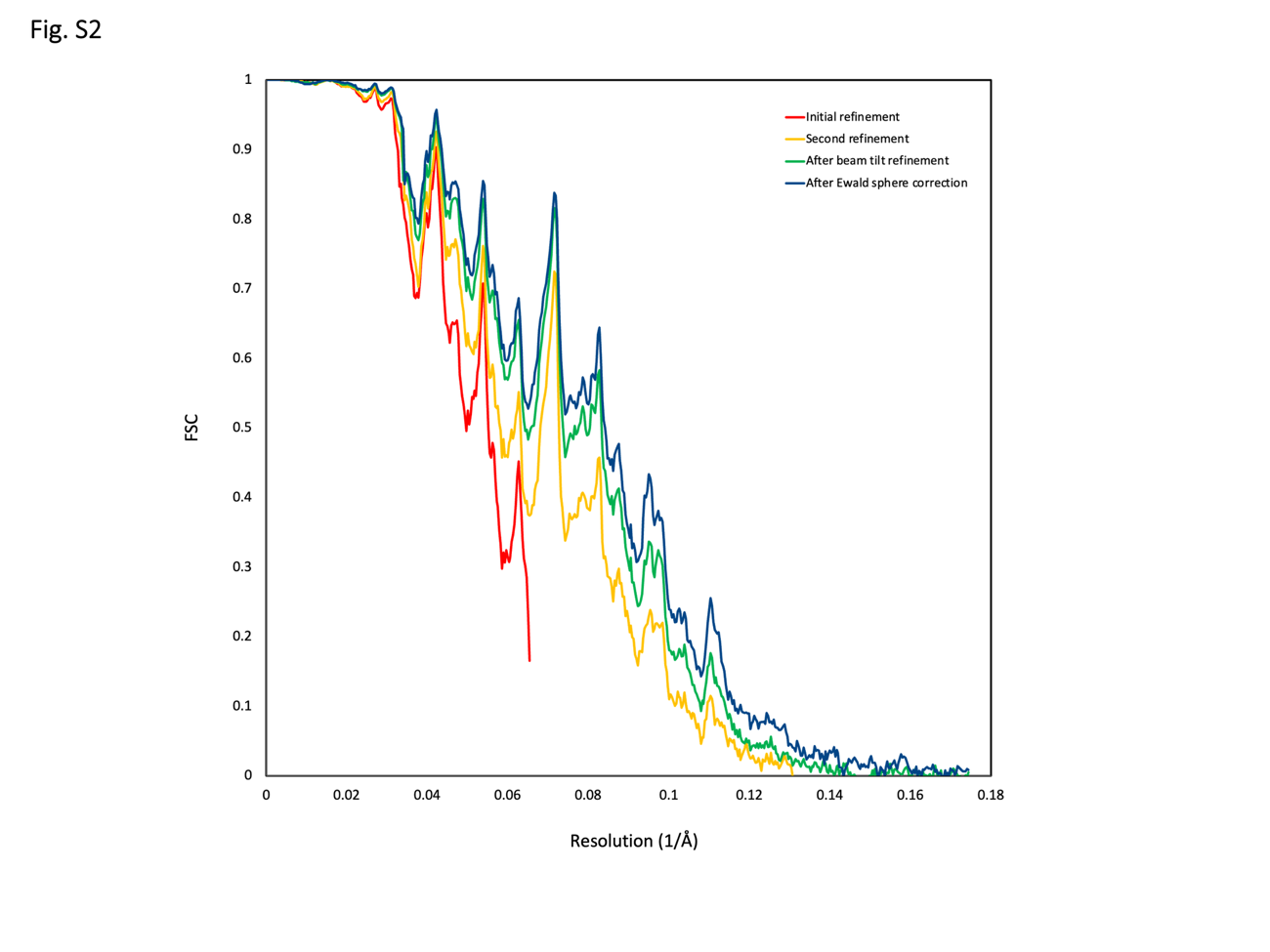


**Figure S2.** Progression of Fourier shell correlation (FSC) curves throughout the refinement process. The initial binned refinement is indicated in red, followed by re-extraction (yellow), further re-extraction and CTF refinement including beam tilt refinement (green), and finally, after application of Ewald sphere correction (dark blue ). Note that while the spiky nature of the FSC remains unchanged, intermediate correlations (i.e., not the spikes of the curve) are significantly improved after beam tilt refinement. Ewald sphere correction had a measurable, but minimal impact at this resolution.

**Movie S1.** Four-hour time-lapse of ushikuvirus-infected cells at 58–62 hpi (MOI of 10). The time-lapse movie was created at 15 frames per second (FPS) from images captured every 15 s.

**Movie S2.** Four-hour time-lapse of uninfected cells. The time-lapse movie was created at 15 frames per second (FPS) from images captured every 15 s.
